# Declarative memory supports children’s math skills: A longitudinal study

**DOI:** 10.1371/journal.pone.0304211

**Published:** 2024-07-25

**Authors:** Tanya M. Evans, Daniel W. Lipscomb, F. Sayako Earle, Stephanie N. Del Tufo, Jarrad A. G. Lum, Laurie E. Cutting, Michael T. Ullman

**Affiliations:** 1 School of Education and Human Development, University of Virginia, Charlottesville, VA, United States of America; 2 Department of Communication Sciences & Disorders, University of Delaware, Newark, DE, United States of America; 3 Peabody College of Education and Human Development, Vanderbilt University, Nashville, TN, United States of America; 4 Department of Education and Human Development, University of Delaware, Newark, DE, United States of America; 5 School of Psychology, Deakin University, Burwood, Victoria, Australia; 6 Department of Neuroscience, Georgetown University, Washington, DC, United States of America; University of Padova, ITALY

## Abstract

Substantial progress has been made in understanding the neurocognitive underpinnings of learning math. Building on this work, it has been hypothesized that declarative and procedural memory, two domain-general learning and memory systems, play important roles in acquiring math skills. In a longitudinal study, we tested whether in fact declarative and procedural memory predict children’s math skills during elementary school years. A sample of 109 children was tested across grades 2, 3 and 4. Linear mixed-effects regression and structural equation modeling revealed the following. First, learning in declarative but not procedural memory was associated with math skills within each grade. Second, declarative but not procedural memory in each grade was related to math skills in all later grades (e.g., declarative memory in grade 2 was related to math skills in grade 4). Sensitivity analyses showed that the pattern of results was robust, except for the longitudinal prediction of later math skills when accounting for stable inter-individual differences via the inclusion of random intercepts. Our findings highlight the foundational role of early domain-general learning and memory in children’s acquisition of math.

## Introduction

Proficiency in mathematics is essential for success in our increasingly technology-focused society [1,2]. Math disabilities, leading to persistent innumeracy in adulthood [3], affect 7–10% of children worldwide [4,5]. For those with difficulties in mathematical skills, early intervention appears promising for improving outcomes [6] Hence, it is of great interest to identify foundational abilities that manifest early and could be reliably used to predict a child’s math skills. Here we focus on typically developing children in order to help lay the groundwork for understanding such abilities, concentrating on specific learning and memory abilities and their relationship with math skills in grades 2 to 4.

Learning math seems to involve memorizing information (e.g., digits, arithmetic facts), acquiring sequential patterns (e.g., the count sequence), and automatizing skills (e.g., in arithmetic). Substantial research has begun to elucidate the neurocognitive bases of these abilities, including contributions of what appear to be domain-specific mechanisms as well as domain-general underpinnings such as executive function and working memory [7–10].

Another promising line of research investigating how math is learned in the mind and brain has begun to examine potential roles of two domain-general learning and memory systems, declarative and procedural memory [11–13]. We focus on these in the present study. Declarative memory is defined here as the learning and memory that rely on the medial temporal lobe and associated circuitry [14]. Procedural memory is defined as the learning and memory that depend on the basal ganglia and associated circuitry [14]. These neurocognitive systems have at least somewhat distinct characteristics as well as neural substrates [14–17]. For example, declarative memory supports the learning of facts, and may be necessary for associating arbitrary pieces of information, whereas procedural memory is better suited for learning and eventually automatizing motor and cognitive skills, including sequences [14–18]. Moreover, the declarative memory system allows one to learn quickly, while the procedural memory system subserves the more gradual acquisition of skills with practice [14–17]. For more on these memory systems, see Refs [14–18]. Note that these neurocognitive memory system conceptualizations differ from some traditional operationalizations of declarative and procedural memory. Traditional views of declarative memory are often centered on the existence of explicit knowledge—rather than our conceptualization of medial-temporal-lobe based learning, whether or not it underlies explicit or implicit knowledge, and indeed evidence indicates that it subserves both [19–21]. Traditional views of procedural memory are often equated with implicit knowledge—rather than our conceptualization of basal-ganglia based learning, which is only one of several implicit learning systems [17,22,23]. Moreover, in mathematical cognition research, procedural memory can refer to operations (“procedures”) as opposed to fact retrieval—even though such terminology refers to operations that may not be implicit and are not necessarily tied to learning in the basal ganglia, and thus are quite distinct from our notion of procedural memory. For discussion see Refs [11,14].

Both of the neurocognitive learning and memory systems, as laid out above, are plausible candidates for contributing significantly to math learning. First, there appears to be overlap between the characteristics of the two systems and those of certain math abilities. For example, learning math involves learning math facts, and declarative memory underlies learning facts. And both learning math and procedural memory involve learning sequences and automatizing skills. Second, there is also overlap between the neural substrates of these systems and those underlying math. The brain network supporting math in children and adults is extensive. It includes regions that support quantity representation (the intraparietal sulcus), object processing (ventral occipitotemporal cortex), and executive control (prefrontal cortex) [24]. Notably, the network also appears to include structures central to both declarative memory (the medial temporal lobe [16,17,25–28] and procedural memory (the basal ganglia [29]), consistent with the mathematical roles of both systems. Finally, the plausibility of the two systems underlying math learning is underscored by the fact that both appear to be broadly domain-general, in that they play learning and memory roles across multiple types of information and modalities [14–17], including other higher cognitive domains such as language and reading [14–17,30] Hence, they may also underlie learning in another domain, namely math.

Indeed, it has recently been hypothesized that both systems underlie the learning, knowledge, and use of mathematical abilities [11–13]. Here we focus on the theoretical framework proposed by Evans and Ullman (2016) [11], namely the declarative/procedural (DP) model of math, which quite simply posits that learning math skills should rely importantly on declarative and procedural memory, specifically, the neurocognitive learning and memory constructs described above.

The DP model is grounded in the notion of co-optation [14,17,31]. According to this basic principle of evolution and biology, previously existing structures and mechanisms are constantly being reused—that is, co-opted—for new purposes. Thus, it is likely that math depends importantly on previously existing neurocognitive substrates that were co-opted for this new function—whether or not those substrates have become further specialized for math through evolution, development, or learning. The DP model of math applies this principle of cooptation to math, with a particular focus on learning—just as the DP model of language applies the principle to learning language [17,31] Indeed, the DP model of math is based on two premises related to learning. First, most of math must be learned, whether or not aspects might be innately specified. Second, declarative memory and procedural memory are arguably the two most important learning and memory systems in the brain, in both animals and humans [17,22,31]. Given these premises and the principle of co-optation, the declarative and procedural memory systems are predicted to play important roles in the learning of math skills. This is the essence of the DP model of math.

Although exactly which aspects of math rely on declarative and/or procedural memory is clearly an empirical question, we may expect both to play a role [11]. For example, since declarative memory underlies learning facts in general, it may also be expected to play a role in learning math facts. Since procedural memory is well-suited for learning and eventually automatizing sequences and other skills in general, aspects of math that share these characteristics may be expected to be learned in this system. Importantly however, the evidence suggests that many cognitive tasks and functions can be supported by both systems, that is, by either system, though often in different ways [14,17,31]. For example, in language, grammar can be learned implicitly to a high degree of automatization by procedural memory but can also be learned (e.g., explicitly) by declarative memory [32]. Which of the two systems underlies which task or function at which point is thought to depend on various factors, including individual differences (e.g., learners who are better at declarative memory should depend more on this system and correspondingly less on procedural memory), learning stage (learning in declarative memory proceeds faster than in procedural memory and so functions that can be learned by both systems tend to be learned first by declarative memory and only later acquired by procedural memory), and development (the two systems have somewhat different developmental trajectories, although learning in both systems seems to be available in children of the ages that are tested in the present study) [14,17,31,32]. Thus multiple factors are likely to affect which of the two memory systems contributes to learning which aspects of math at which point during learning in which individuals at which ages.

So how can one test the predictions of the DP model of math? As with the DP model of language, a wide range of behavioral and neurocognitive approaches can be used [11,14,17,31–34]. Here we focus on a specific methodological approach that is particularly effective at revealing links between math and the learning and memory systems that may underlie it [32,33]. This “correlational approach” simply examines correlations (associations) between how well participants learn or process math and how well they learn in the memory systems. Thus, the correlational approach leverages individual differences both in math abilities and in learning abilities in the memory systems to test for links between them. For example, if aspects of math are learned by declarative memory, then an independent measure of learning in this system should predict those math abilities. The correlational approach may be a particularly valid test of the predictions of the DP model since, as we have seen, declarative and procedural memory are operationalized as the learning and memory abilities that depend on particular neural circuitry. Therefore, testing associations between math abilities and these learning abilities—that is, learning performance in tasks that have been independently linked to the neural circuitry of one or the other memory system—can directly test the model’s predictions [32,33]. In contrast, linking math abilities to the neural bases of one or the other memory system (e.g., to the neostriatum) may constitute a weaker test of the reliance of math on the memory system, since those neural bases are not necessarily dedicated to learning and memory (e.g., the neostriatum has many functions apart from procedural memory) [32–34].

Nevertheless, the correlational approach must be used cautiously, since correlation does not imply causation. For example, a correlation between math and declarative memory abilities could be explained not only by math being learned in this memory system but instead or in addition by some other cognitive process that underlies or affects both math and declarative memory. There are various ways that one can address this problem [34]. These include minimizing the involvement of these other processes in one’s task (e.g., minimizing working memory involvement in a task probing declarative memory, and thus selecting tasks and measures that are relatively process-pure), and attempting to hold these processes constant if one can identify them (e.g., covarying out potentially confounding variables in statistical analyses). In the present study, we use both of these approaches.

The goal of the present study is to test whether learning abilities in the declarative and procedural memory systems are associated with and indeed predict children’s math abilities during elementary school years. We emphasize two features of our experimental design. First, we tested children during a known key stage of learning mathematics (8–10 years old), when arithmetic proficiency is rapidly increasing [25]. During this developmental stage there is substantial individual variability in math ability, with some children making significantly greater gains than others [24]. Additionally, at these ages these learning and memory systems seem to be reasonably well developed [17,32,35]. Thus, this age range seems to be an appropriate starting point for investigating whether declarative and/or procedural memory can help explain individual differences in math skills. Second, we employed a longitudinal design, which allowed us to characterize individual differences in skill levels across a developmental time course. We were thus able to examine the temporal relationship between learning abilities in each system and math skills, in order to examine whether learning in the declarative and/or procedural memory systems at one timepoint are in fact predictive of math skills at a later timepoint. This feature of our study speaks to the potential to predict math disability, which may be investigated in future research.

Specifically, we examined a longitudinal cohort of elementary school children, who were tested after completion of 2^nd^ grade, and then again at two further timepoints, following completion of the 3^rd^ and 4^th^ grades. Children’s learning abilities in both memory systems were tested with well-established tasks that are relatively process-pure and have high validity. To capture the roles of these learning and memory systems across various aspects of math at these stages of development, we acquired standardized measures of mathematics achievement. Thus, this study does not attempt to identify exactly which aspect of math each memory system may subserve at which time point during learning. Rather, it takes a foundational approach of investigating whether the memory systems may underlie a wide range of math abilities, to reveal whether in fact the systems may broadly subserve math. Assuming a positive result regarding roles for one or both memory systems, future work should follow up on this study by focusing on particular aspects of math, as well as more fine-grained questions such as how each memory system may be involved during the time course of learning each aspect of math.

In brief, a longitudinal data set was used to investigate: (1) whether learning abilities in the declarative and/or procedural memory systems were associated with a broad measure of math skills within grades 2, 3, and 4; and (2) whether learning abilities in either or both systems at each grade predicted math skills in later grades.

## Material and methods

### Participants

Children from schools in the greater Nashville area were recruited for this study. Families were screened via phone enrollment. Participants’ parents reported no history of major psychiatric illness, traumatic brain injury, or epilepsy. Children had normal or corrected-to-normal vision and were native speakers of American English. Written parental consent and informed child assent were obtained in compliance with the Vanderbilt University Institutional Review Board. All participants received compensation for their participation. Recruitment for the study began in June of 2016, and participants came in for data collection between August 2017 and May 2018. Dr. Cutting is the only author who had access to information that could identify individual participants, but she was not directly involved in data analysis.

Data were acquired as part of a larger study at the Education & Brain Science Research Lab at Vanderbilt University. Here, we present the data collected on math ability, declarative memory, and procedural memory following completion of grades 2–4. A total of 109 children (61 females, 48 males) participated at a minimum of one timepoint. A sensitivity analysis examining only children for which we had the key learning and math measures at all three grades (n = 61) showed the same pattern of results as the main analyses carried out on the full set of children; see Results.

Children completed standardized assessments of mathematics as well as experimental tasks probing learning in declarative memory and learning in procedural memory. All tests were administered in the summer after each school year (i.e., 2^nd^, 3^rd^, and 4^th^ grade). Trained experimenters administered all assessments in a quiet room. The experimental tasks were presented on the LCD screen of a PC computer, using E-Prime 2.0 (Psychology Software Tools) to control stimulus presentation and response recording. Responses were recorded via button press either on a serial response box (declarative memory task) or a number pad (procedural memory task).

### Mathematical ability

Children’s math ability was assessed with the Calculation subtest of the Woodcock-Johnson Test of Achievement 3^rd^ Edition [36], which measures the ability to perform basic computations ranging from writing numbers through solving arithmetic problems (i.e., addition, subtraction, multiplication, division), as well as more advanced operations depending on the student’s ability (i.e., geometric, trigonometric, logarithmic, and calculus). Raw scores were subjected to data transformations, described below.

### Declarative memory

A non-verbal recognition memory task developed at the Brain and Language Lab at Georgetown University was used to assess declarative memory [37,38]. Such tasks are commonly used to probe declarative memory, and, crucially, are closely tied to its medial temporal lobe substrates, suggesting that the task has high validity, given the neurocognitive conceptualization of this memory system. The task’s validity is further strengthened by evidence indicating that performance on the task correlates with a distinct declarative memory task, and loads on the same factor as that task in a factor analysis [39]. Moreover, the task is an incidental encoding recognition memory task, which directly assesses declarative memory while minimizing the involvement of working memory, recall, and phonology [37,38]. Thus, the task is also relatively process-pure.

The stimuli were black and white line drawings of 64 real and 64 made-up objects. Images of real objects were taken from various sources, then modified as necessary. Sources included clip art galleries (including free websites and purchased collections) and the Snodgrass and Vanderwart [40] set of pictures. Images for made-up objects were taken from Cornelissen et al. [41], Eals & Silverman [42] and Williams & Tarr [43], then modified as necessary. All images were sized, touched up, rotated, and/or converted to black and white to create the final set of stimuli.

During the incidental encoding phase, the children were asked to indicate whether each object was real or made-up (an object decision task). They placed their index fingers on the left and right keys of the serial response box, and pressed either the left or right key to indicate if images were real or made-up, respectively. To remind children which response corresponded to each key, “real” was displayed in the bottom left corner of the screen and “made-up” in the bottom right corner. The children were asked to respond as quickly and as accurately as possible. They were given 3 practice trials followed by 64 experimental trials (50% real objects, 50% made up objects). The stimuli were pseudorandomized; no trial type was repeated consecutively more than three times. In each trial, a centrally placed fixation cross was presented for 1000ms, after which each item was displayed on the screen for 500ms, irrespective of when the response occurred, thus controlling for the duration of stimulus exposure during encoding. If a child responded within 500ms of stimulus presentation, the trial ended immediately after the 500ms. If a child did not respond during this period, a fixation cross then appeared, for up to 4500ms or until the child responded. If a child still did not respond during this additional period, the trial ended. Following each trial a buzzer sounded for 200ms, followed by 800ms of fixation before the next trial.

After a ten-minute break, the recognition phase was presented. Children were asked to indicate via button press whether or not they had previously seen the object presented in the center of the screen. They again placed their index fingers on the left and right keys of the serial response box, and pressed either the left or right key to indicate if images had or had not been seen before, respectively. To remind children which response corresponded to each key, the question “seen before?” was shown in the bottom middle of the screen, and “yes” and “no” were displayed in the bottom left and right corners, respectively. Children were asked to respond as quickly and accurately as possible. They were given six practice trials followed by 128 experimental items (50% real, 50% made up). Half the real and half the novel objects in the experimental items had been seen during the encoding phase, while the other half were foils that were not seen during encoding. Real object stimuli were matched for word frequency, number of syllables, and number of phonemes between the target items (presented during both the encoding and recognition phases) and the foils (presented during the recognition phase). The timing parameters for the recognition phase were identical to the encoding phase.

### Procedural memory

Nissen and Buellemer’s [44] non-verbal serial reaction time (SRT) task (modified as in Ref [45]) was used to assess procedural memory. The SRT is a widely-used task that probes sequence learning in procedural memory, and is closely tied to the neural substrates of procedural memory, in particular the basal ganglia, via both lesion and neuroimaging studies [46,47]. Indeed, a recent functional neuroimaging meta-analysis revealed selective activation in the basal ganglia for implicit sequence learning in the SRT [47], specifically in the anterior neostriatum, which has been strongly implicated in procedural learning [14]. This suggests that the task not only has high validity (that is, learning is closely tied to the basal ganglia, in particular the anterior neostriatum) but also is relatively process-pure (that is, it is tied selectively to the basal ganglia, in particular the anterior neostriatum).

In this task a yellow smiley face (from a Clipart gallery) appeared in one of four locations arranged in a diamond pattern on the screen. Children used their dominant hand to press marked buttons on the number pad (2, 4, 6, 8) corresponding to the location of the smiley face on the screen. Children completed 10 practice trials followed by 360 experimental trials in six blocks of 60. In blocks 1 and 6, the stimuli were presented in a pseudorandom sequence. In blocks 2–5, an identical sequence of 10 locations was repeated 6 times in each block. Children were asked to respond as quickly and accurately as possible.

### Data transformation

As is standard in recognition memory tasks, accuracy scores in the task were transformed to *d’* scores in order to account for potential response biases (see Ref [48]). Each such score was calculated as the *z*-score of the hit rate minus the *z*-score of the False Alarm rate; a higher *d’* score indicates higher performance. As in previous pediatric and other research employing the SRT task [45], the procedural memory score was calculated as the difference between the average RT on correct responses in Block 6 (random) and the average RT on correct responses in Block 5 (sequence) for correct trials. Greater scores are taken to indicate better performance, in particular the extent to which sequence knowledge was acquired during the task. These standard dependent measures of learning in declarative memory and procedural memory are well-validated. For example, the functional neuroimaging meta-analysis mentioned above that linked sequence learning in the SRT task to the anterior neostriatum was based on studies using such differential response times between sequence and random blocks.

Furthermore, we scaled the raw math scores, the *d’* scores from the declarative memory task, and the procedural memory scores from the SRT task, using Proportion of Maximum Scaling. Specifically, the observed scores were transformed in proportion to the lowest and highest possible scores of each measure. Thus, for all three tasks 0 was the minimum score and 1 was the maximum score following scaling. Proportion of Maximum Scaling ensures that differences in scores between timepoints and individuals are in correct proportion, and transforms variables to commensurate scales, while at the same time conserving the underlying distribution and covariance matrix of variables. This method has been found to be particularly useful for longitudinal data sets [49].

### Statistical analyses

#### Within-grade relationship between learning abilities and math ability: Mixed-effects models.

To test the within-grade relationship between the children’s declarative memory and procedural memory abilities on the one hand, and their math abilities on the other, across the 2^nd^, 3^rd^ and 4^th^ grades, we forward-fitted a series of linear mixed-effects models with robust standard errors, in which child scores by-grade were nested within participants. Starting with a model that included the between-person random intercept to correct for the nesting of observations within children, we sequentially added the following fixed effects in increasingly complex models: (1) grade 2 and 4 indicators (with grade 3 as the comparison group), as well as child age and sex as covariates to control for variability in these factors; (2) declarative and procedural memory scores; and (3) interaction terms between the memory variables and grade (as well as age and sex) to test for developmental differences in math ability. We used information criteria (AIC and BIC) among these models to select the best fitting model. After the case-wise deletion of observations that were missing any memory scores (that is, any grade for any child for which declarative and/or procedural memory scores were missing; note that observations for which math ability scores were missing had already been excluded as this is the outcome measure), the mixed-effects models included 192 child-grade observations nested in 97 participants. See Table 1 for the descriptive statistics and normality tests for the full data set, that is, before case-wise deletion. For further explanation of the nesting structure, information criteria, and missing scores, see Supporting (Supplementary) Information.

**Table 1:** Descriptive statistics for child-grade observation data, for mixed-effects modeling.

Variables	*M*	*SD*	Skewness	Kurtosis	Shapiro-Wilk *p*	Individual ICC [95% *CI*]
Grade ^a^	2.95	0.81	0.08	1.52	*p ≈* 1.00	-
Sex ^b^	0.46	0.50	0.16	1.02	*p ≈* 1.00	-
Age	9.39	0.88	0.04	1.91	*p* < .001	-
DM	0.49	0.18	-0.26	2.81	*p* = .07	.44 [.29, .60]
PM	0.58	0.14	-0.18	4.48	*p* = .04	.32 [.08, .58]
Math	0.53	0.20	0.11	2.42	*p* = .09	.66 [.54, .76]

*Note*. *M* refers to Mean, *SD* to Standard Deviation, ICC to Intraclass Correlation (a consistency-of agreement measure of reliability for DM, PM, and Math within a participant across grades), *CI* to Confidence Interval, DM to Declarative Memory scores, PM to Procedural Memory scores, and Math to Mathematical ability scores. Grade and Sex are categorical variables, while the remaining variables are continuous. DM, PM, and Math were scaled using Proportion of Maximum Scaling. The data are in long format, such that 261 individual observations contain the children’s measurement instances for each grade (i.e., each of the 109 children can have up to three observations, one per grade); however, the mixed models only included 192 observations from 97 subjects after case wise deletion of cases with missing DM and/or PM scores; see Methods. Several variables violated normality assumptions according to the Shapiro-Wilk test, and thus we employed robust standard errors for all our mixed-effects models.

^*a*^ 92 observations were from grade 2, 89 from grade 3, and 80 from grade 4.

^*b*^ 141 observations were female (coded as 0 in the table), 120 were male (coded as 1).

### Learning ability predicting later math ability: Structural equation models

To test the predictive value of declarative and procedural memory in earlier grades on math ability in later grades, we examined indirect effects using time-series Structural Equation Models (SEMs) with within-person centered [50] memory and math ability variables at each grade, and robust standard errors [51]. We modeled predictive pathways for memory influencing math ability both within a grade (e.g., declarative memory predicting math ability within grade 3) and between-grades (e.g., declarative memory in grade 3 predicting math ability in grade 4), and also included all autoregressions to capture the relationship of each variable to itself in the subsequent grade (e.g., declarative memory in grade 2 to declarative memory in grade 3, or math ability in grade 3 to math ability in grade 4). Initial analysis showed that the memory variables related to math ability more within a grade than between grades, such that it would be difficult to distinguish if memory in one grade would have a causal effect on math ability in the next grade, or if this relationship would be mediated by processes at a smaller time-scale within a grade. For instance, higher declarative memory could be instrumental to successful math learning and achievement for a child within a given grade, with the mathematical knowledge gained in that grade then serving as the foundation for math ability in the next grade. Based on this nature of the data, we decided to test indirect effects instead of cross-lagged effects.

**Fig 1:**
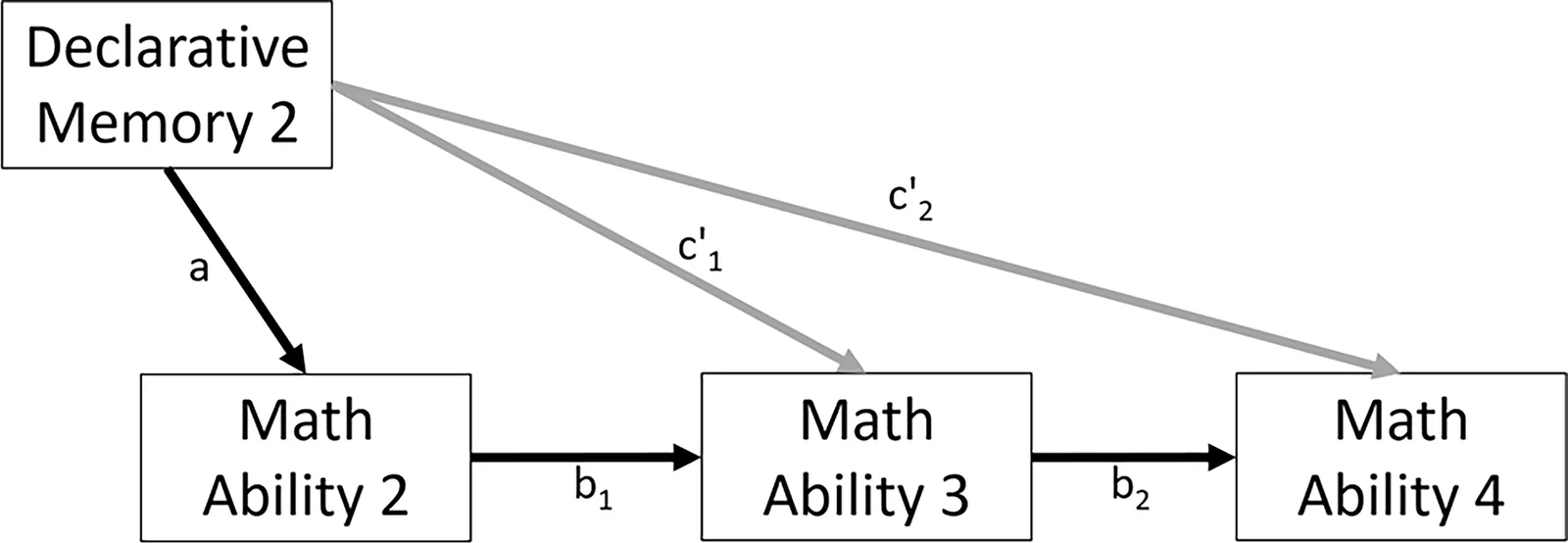
Example of an indirect effect pathway. The number at the end of the variable name refers to grade 2, 3, or 4. Black directional lines indicate significant relationships (p < .05), while gray lines indicate non-significant relationships. In this example, while declarative memory in grade 2 does not directly relate to math ability in grade 4 (i.e., the c’_2_ path), it can be shown to indirectly relate to grade 4 math ability through its direct relationship with math ability in grade 2 (path a), math ability in grade 2 relating to math ability in grade 3 (path b_1_), and math ability in grade 3 influencing math ability in grade 4 (path b_2_). Statistically, this indirect effect would be supported if the product of coefficients a, b_1_, and b_2_ was significantly different from zero. Also, note that the a and b paths cannot be estimated without also including the c’ paths, which are the residual direct effects of a predictor on the outcomes.

We then tested the statistical indirect effects (i.e., the product of regression coefficients) for various combinations of these pathways (declarative/procedural memory within grade, math ability between grade; see Fig 1 for a visual example). In all models, declarative and procedural memory freely covaried within each timepoint. All available information from the 109 children was retained, using Full Information Maximum Likelihood (FIML).

The model iterations started with an autoregressive model, in which regressions from grade 2 to 3 and grade 3 to 4 were calculated within each variable. In this and all subsequent models, sex as well as age for that grade were included as covariates for math ability. Next, we introduced paths for which memory variables predicted math ability within the same grade (e.g., declarative memory predicts math ability in grade 4) and in the next grade (e.g., procedural memory in grade 3 predicts math ability in grade 4). We used a Satorra-Bentler scaled chi-square difference test to see if the predictive paths improved model fit over the autoregressive model; we did not find any instances of the Satorra-Bentler test being used with bootstrapped models in the literature, so we compared non-bootstrapped models to ensure the validity of model comparison. Finally, we estimated the cross-variable model (i.e., in which the relationships between memory and math are included) using 10,000 bootstrap resamples to obtain our final model parameters and significance tests of the indirect effects using bias-corrected confidence intervals (*CI*s). We computed not only 95 percent *CI*s, but also more conservative 99 percent *CI*s; see Results, and Supporting Information.

See Table 2 for the descriptive statistics and normality tests for these data; note that Tables 1 and 2 are both based on the full data set, but show different information, corresponding to the mixed-effects and SEM analyses, respectively. Table 3 shows all pairwise correlations for all available data for each pair of variables. See Supporting Information for more details about FIML, the Satorra-Bentler test, and bootstrapping, as well as for a discussion on small sample size concerns.

**Table 2:** Descriptive statistics for child-level longitudinal data, for structural equation modeling.

Variables	*M*	*SD*	Skewness	Kurtosis	Shapiro-Wilk *p*
Sex ^a^	0.44	0.50	0.24	1.06	*p ≈* 1.00
Age 2	8.44	0.34	-0.14	2.05	*p* = .21
Age 3	9.43	0.33	-0.25	2.22	*p* = .14
Age 4	10.44	0.33	-0.16	2.32	*p* = .48
Math 2	0.37	0.12	-0.40	3.41	*p* = .58
Math 3	0.55	0.15	-0.38	2.96	*p* = .24
Math 4	0.69	0.18	-0.52	2.43	*p* = .01
DM 2	0.47	0.18	-0.40	2.68	*p* = .08
DM3	0.48	0.18	-0.25	2.88	*p* = .57
DM4	0.53	0.17	-0.09	2.59	*p* = .19
PM 2	0.56	0.14	0.34	4.15	*p* = .22
PM 3	0.56	0.13	-0.85	6.30	*p* = .002
PM 4	0.62	0.14	-0.07	3.21	*p* = .74

*Note*. *M* refers to Mean, *SD* to Standard Deviation, DM to Declarative Memory, PM to Procedural Memory, and Math to Mathematical ability scores. The number at the end of a variable refers to grade (i.e., grade 2, 3, or 4). DM, PM, and Math were scaled using Proportion of Maximum Scaling. Several variables violated normality assumptions according to the Shapiro-Wilk test, and thus non-normality corrections were employed in our models.

^*a*^ 61 children were female (coded as 0 in the table), 48 were male (coded as 1).

**Table 3:** Pairwise correlations of transformed variables, within and across grades.

Variables														
	*n*													
*r*		Sex	Age 2	Age 3	Age 4	Math 2	Math 3	Math 4	DM 2	DM 3	DM 4	PM 2	PM 3	PM 4
Sex		109	92	89	80	92	89	80	88	87	79	48	83	67
Age 2		0.20	92	74	68	92	74	68	88	72	68	48	68	57
Age 3		0.20	0.97***	89	75	74	89	75	71	87	75	40	83	63
Age 4		0.24*	0.96***	0.96***	80	68	75	80	64	74	79	38	71	67
Math 2		0.04	0.19	0.08	0.11	92	74	68	88	72	68	48	68	57
Math 3		0.12	0.08	0.05	0.15	0.62***	89	75	71	87	75	40	83	63
Math 4		-0.03	-0.12	-0.10	-0.11	0.60***	0.74***	80	64	74	79	38	71	67
DM 2		-0.07	-0.05	-0.03	0.09	0.28**	0.27*	0.25*	88	69	64	45	65	54
DM 3		0.05	0.12	0.01	0.16	0.25*	0.36***	0.32**	0.63***	87	74	38	81	62
DM 4		0.03	0.11	0.14	0.12	0.25*	0.32**	0.41***	0.38**	0.43***	79	38	71	66
PM 2		0.25	0.19	0.18	0.24	-0.03	-0.14	-0.06	0.12	-0.22	0.08	48	38	29
PM 3		0.03	0.01	0.04	0.03	-0.04	-0.04	-0.03	-0.09	-0.09	0.01	0.29	83	59
PM 4		-0.03	0.01	0.05	-0.01	-0.05	-0.02	-0.07	0.17	0.13	0.27*	0.16	0.13	67

*Note*. * p < .05, ** *p* < .01, *** *p* < .001. *N =* 109. Math refers to Mathematical achievement scores, DM to Declarative Memory, and PM to Procedural Memory. The number at the end of each variable refers to grade (i.e., grade 2, 3, or 4). Cells in the bottom left of the table present the pairwise correlation coefficient *r*, while cells in the top right show the pairwise sample for each correlation. Based on the prevalence of missing data, we used Full Information Maximum Likelihood to retain all available information in our Structural Equation Models; see Methods.

*Software*: Data management and mixed-effects modeling were handled in STATA 15.1 [52]. Structural Equation Models were run in MPlus 8.4 [51].

*Data availability*: The datasets analyzed during the current study are available from the corresponding author on reasonable request.

## Results

### Learning in declarative memory relates to within-grade math ability

The mixed-effects models tested whether learning abilities in declarative and/or procedural memory related to within-grade math ability, examined over all grades. Our forward-fitting approach suggested that the model with the two memory predictors, but not with additional interaction terms with grade, age, or sex, showed the best fit, in terms of AIC and BIC (see S4 Table in S1 Text). This model revealed that declarative memory was significantly related to within-grade math ability, over all grades (*b* = 0.20, *SE* = 0.06, *p* < .001); specifically, higher declarative memory was associated with higher math abilities. As expected, this model also revealed that (independent of declarative memory abilities) math abilities improved across subsequent grades: compared to math ability in grade 3, achievement was significantly lower in grade 2 (*b* = -0.16, *SE* = 0.05, *p* < .001) and higher in grade 4 (*b* = 0.12, *SE* = 0.05, *p* = .01). No other fixed effects in the model were significant. Most importantly, the results from this model suggest that declarative memory (but not procedural memory) was related to within-grade math ability across grades 2 to 4, even when accounting for a child’s procedural memory, grade, age, and sex. See S4 Table in S1 Text for all model comparisons and results.

### Learning in declarative memory predicts later math ability

The SEMs tested whether declarative and procedural memory abilities in a given grade predicted later math ability, accounting for age and sex. Compared to the autoregressive model (the base model, which included only the relationship of each variable to itself in the following grade), a model with the addition of pathways in which declarative memory predicts math ability both within grades and across grades (e.g., grade 2 to 3 or grade 2 to 4) significantly improved model fit (*Satorra-Bentler* Δ*χ*^2^ = 30.60, Δ*df* = 9, *p* < .001). Procedural memory did not relate to math scores, so was only retained as a covariate. After bootstrapping, the final model showed excellent fit (*CFI* = .97; *RMSEA* = .03; Ref [60]).

**Fig 2:**
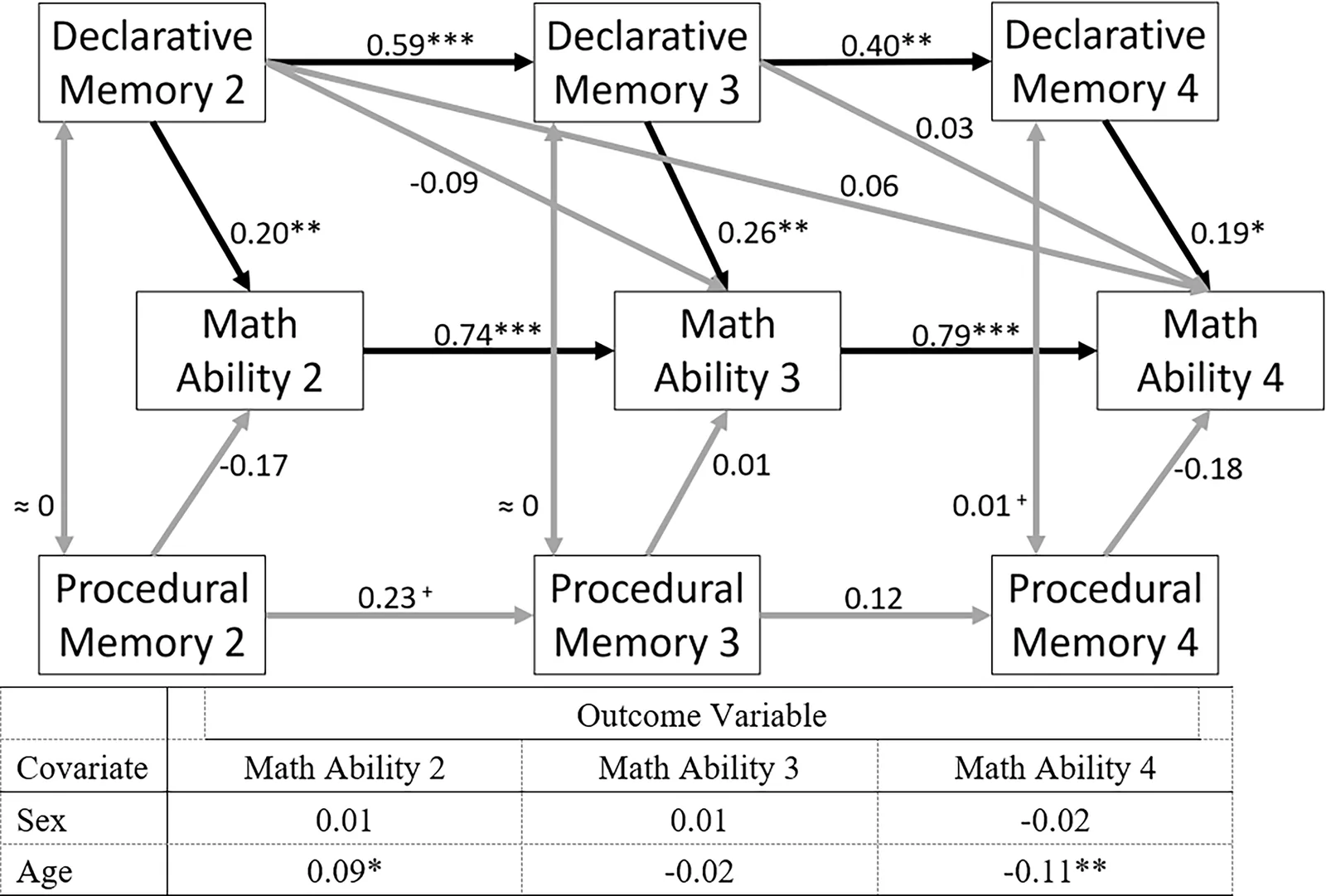
Final SEM model, following bootstrapping. CFI = .97, RMSEA = .03, AIC = -1009.12, n-adjusted BIC = -1035.82, N = 109. The number at the end of each variable name refers to grade 2, 3, or 4. Lines with single arrows represent regressions, while lines with double-arrows (between declarative memory and procedural memory) show covariances. Significant paths are shown in black, while non-significant ones are gray. Unstandardized coefficients (b) are presented adjacent to the path lines. Data for the memory and math ability variables were transformed using Proportion of Maximum Scaling, and were person-centered for each grade; see Methods. The regressions for math scores included sex as well as age for that grade as covariates; these coefficients are presented below the figure. ^+^ p < .10, * p < .05, ** p < .01, *** p < .001.

The direct effects in the final model revealed, first of all, that both declarative memory and math abilities were consistent over time, in that higher scores for each variable in one grade significantly predicted higher scores for that variable in the next grade (for example, a child with high math abilities in grade 2 would likely have high math abilities in grade 3); however, there were no such autoregressive relationships for procedural memory. Moreover, consistent with the results from the mixed-effects model presented above, declarative memory in grades 2, 3, and 4 significantly predicted math ability within the same grade (Grade 2: *b* = 0.20, *SE* = 0.06, *p* = .002; Grade 3: *b* = 0.26, *SE* = 0.09, *p* = .002; Grade 4: *b* = 0.19, *SE* = 0.09, *p* = .04). No other effects of interest were significant. See S4 Fig in S1 Text for a depiction of the autoregressive model, and Fig 2 for the final model.

The indirect effects show that declarative memory in each grade significantly predicted math ability in all later grades; see Fig 2 and Table 4. That is, declarative memory abilities in grade 2 indirectly predicted math abilities in grade 3 and grade 4, while declarative memory in grade 3 predicted math in grade 4. In all cases these indirect relationships were obtained through two or more indirect pathways (that is, through two or more distinct mediators). For example, grade 2 declarative memory indirectly predicted grade 3 math through two different indirect paths (that is, through two different mediators, grade 2 math and grade 3 declarative memory). To put it differently, each indirect effect of declarative memory to later math abilities involved one or more autoregression direct effects (e.g., declarative memory in grade 2 predicting declarative memory in grade 3, or analogously for math) as well as one direct effect of declarative memory predicting math in the same grade. See Fig 2. All the indirect paths were significant at 95% confidence intervals; see Table 4.

**Table 4:** Indirect effects of sex & age covariate model.

Outcome	Indirect Pathway	95% Bias-Corrected Confidence Interval	99% Bias-Corrected Confidence Interval
Math Ability 3			
	DM 2 → DM 3 → Math 3	[0.06, 0.30]	[0.03, 0.35]
	DM 2 → Math 2 → Math 3	[0.06, 0.25]	[0.03, 0.29]
Math Ability 4			
	DM 3 → DM 4 → Math 4	[0.01, 0.18]	[-0.01, 0.22]
	DM 3 → Math 3 → Math 4	[0.07, 0.36]	[0.02, 0.42]
	DM 2 → DM 3 → DM 4 → Math 4	[0.01, 0.12]	[0.00, 0.15]
	DM 2 → DM 3 → Math 3 → Math 4	[0.05, 0.24]	[0.02, 0.29]
	DM 2 → Math 2 → Math 3 → Math 4	[0.05, 0.21]	[0.02, 0.25]

*Note*. Any confidence interval that did not contain zero was considered significant. Parameter estimates were taken from a bootstrapped model with 10,000 resamples. DM refers to Declarative Memory Scores and Math to Mathematical ability scores. The number at the end of the variable name refers to grade 2, 3, or 4. The regressions for math scores included sex as well as age for that grade as covariates. See Fig 2 for a visual representation of the direct effects of this model.

When using a more conservative 99% confidence interval all indirect relationships were still maintained (that is, in every case declarative memory in one grade predicted math at all later grades), though not all *specific* indirect pathways maintained significance. In particular, due to a somewhat weak direct effect of declarative memory in grade 4 to math abilities in grade 4, indirect paths including this effect were not significant at a 99% confidence interval; see Table 4. Nevertheless, we emphasize that even with a 99% confidence interval all other indirect pathways were still significant, and thus every indirect relationship between declarative memory abilities and later math abilities still held at this more stringent confidence interval.

To test the robustness of the indirect effects we also performed three sensitivity analyses. In all three cases the sensitivity analysis involved one or more specific changes to the final SEM model described above. In the first sensitivity analysis we ran a model with no covariates (that is, sex and age were removed), since the inclusion of covariates can substantially change regression estimates [53,54]. In the second sensitivity analysis, the model included only children who had complete data for both declarative memory and math ability (n = 61), that is, children for whom we had both of these measures from all three grades. This was designed to address potential concerns regarding the presence of missing data for some participants in some grades in the main analysis described above. The third sensitivity examined the same restricted sample of 61 participants, but excluded procedural memory from the model, since this variable yielded the most missing data. Importantly, all three sensitivity analyses yielded the same pattern of results produced by the main model, namely declarative memory abilities in earlier grades significantly predicted (at both 95% and 99% confidence intervals) math abilities in later grades via one or more indirect pathways, including from grades 2 to 3, grades 2 to 4, and grades 3 to 4. See S1–S3 Figs and S1–S3 Tables in S1 Text.

Finally, we attempted to run our main SEM with the inclusion of longitudinal random intercepts [55] for each set of variables (e.g., Math 2, Math 3, and Math 4), to control for stable differences within these measures across timepoints [56]. This model design helps to separate the variation between timepoints that is shared across subjects from the variation *within* a subject, such that we can better test if a subject’s increase in declarative memory or procedural memory at an earlier timepoint predicts their own increases in math at a later timepoint, which provides stronger causal evidence. Unfortunately, due to difficulties in estimating this longitudinal random intercept SEM with our data, we could only estimate it with the correlation matrix of our variables. These models produced null results for all our previously reported single-mediator indirect effects (e.g., DM 3 → DM 4 → Math 4), and we were not able to test the two mediator indirect effects (e.g., DM 2 → DM 3 → Math 3 → Math 4). However, given general estimation issues in testing this random intercept model directly, and the results of a power analysis to find indirect effects in a random intercept model given our data, which suggested that these analyses were underpowered, we cannot be sure whether there was in fact no subject-specific relationship between our variables, or if instead there was no adequate means of testing it with random intercepts given our data. Nevertheless, we recognize that the potential underpowering of the study to detect the effect of interest when employing this more suitable analysis is a limitation inherent to the study design rather than the analytical plan per se. See Supporting Information for further details. Thus, caution is warranted in interpreting the indirect effects, and future studies investigating this issue seem desirable.

## Discussion

In this longitudinal investigation, we evaluated whether elementary school children’s learning abilities in the declarative memory and/or procedural memory systems predict their math abilities. Linear mixed-effects regression and structural equation modeling showed the following. First, learning abilities in declarative but not procedural memory were associated with math ability within each grade, across grades 2, 3, and 4. Second, evidence suggests that learning abilities in declarative but not procedural memory in each grade may be related to math abilities in all later grades that were examined. Overall, the findings provide novel evidence for a foundational role of declarative memory in the development of children’s math skills.

### Learning in declarative memory relates to within-grade math ability

Both the mixed-effects regression and SEM analyses showed that declarative memory is strongly associated with math ability within the same grade. This relationship was found in elementary school children in the 2^nd^, 3^rd^ grade, and 4^th^ grades; moreover, the extent of this relationship did not differ among the grades.

This pattern is consistent with the DP model of math as well as with prior research. In particular, previous studies have suggested a developmental shift from a very early reliance on immature strategies such as counting to an increased reliance on putative memory-based retrieval strategies [57], as children’s math abilities improve. The findings presented here support such a reliance, and directly implicate declarative memory. Indeed, at the brain level, evidence suggests that this maturational shift in strategy is accompanied by an increased reliance during arithmetic processing on the hippocampus [25], the neuroanatomical core of the declarative memory system [32,58]. Note also that an increasing mathematical dependence on declarative memory is consistent with independent evidence indicating that declarative memory matures over the course of childhood [17].

While the exact mechanistic role of declarative memory in math learning is not well understood, our results provide an important step forward in its characterization. We suggest two non-mutually exclusive possibilities to help contextualize our findings: (1) declarative memory may be contributing to the learning and retention of mathematical information, perhaps particularly that which is taught in classroom settings (consistent with evidence that explicit knowledge is learned in this system [32]); and/or (2) this medial temporal lobe memory system may be involved in supporting mathematical operations, for example by way of its involvement in working memory [59]. Future research could resolve these possibilities by testing the role of declarative memory in specific numerical operations.

### Early learning abilities in declarative memory may be related to later math skills

The structural equation modeling suggested that learning abilities in declarative memory in each grade may predict math abilities in all later grades. For example, learning in declarative memory in grade 2 was associated with math abilities not only in grade 3 but even in grade 4. Nevertheless, caution is warranted given that analyses including a longitudinal random intercept were inconclusive due to insufficient power. Overall, these results suggest that the declarative memory system might constitute a foundation for the successful acquisition of math. In particular, it appears that better declarative memory during the early years of math education might play a role in stronger mathematical abilities in somewhat later years. These findings not only suggest the possible importance of declarative memory in math learning, but also argue for the promise of this memory system as a potential early target in both typical learning contexts and in cases where mathematical deficits are suspected. Nevertheless, we emphasize that further studies are needed.

### Differential contributions of declarative and procedural memory systems

Our final result of note from both the mixed-effects and SEM analyses is that learning abilities in procedural memory were not associated with math ability, even while declarative memory abilities were. The differential involvement of these memory systems in the acquisition of math during early years of education (that is, during elementary school) is of particular interest. The notion that declarative memory is a foundational skill in younger children, while procedural memory plays less of a role during these early years, even while independent evidence implicates this system in math learning [11], is consistent with both the memory and math literatures. First, it jibes with the fact that learning in declarative memory can be quite rapid, whereas in procedural memory learning and eventual automatization occurs gradually with practice [14–17]. Second, it fits with evidence suggesting that different math abilities may be required for success at different stages in mathematical education. Learning math is initially slow and effortful as children have to perform various memorized arithmetic functions in a step-by-step manner; yet with increased practice, the processes become automatized, and procedural memory may contribute or take over specific computational roles [11]. Interestingly, the differential involvement of declarative and procedural learning at earlier versus later stages also aligns with evidence indicating the same pattern in second language acquisition [33,60]. Moreover, it is consistent with a recent finding in the domain of reading acquisition that showed an early reliance on declarative memory, with procedural memory involvement coming In later during development [30]. These converging lines of evidence support the validity of the findings reported here. Further research investigating the role of both memory systems in older children could test the hypothesis that procedural memory does in fact play important roles during typical math learning [11], but mainly later in the course of development.

### Limitations and future directions

This study’s limitations as well as findings suggest new lines of research, in addition to those suggested above. First, although the tasks and measures used to probe declarative and procedural memory are well-motivated, have high validity, and are relatively process-pure (see Methods), other tasks assessing these memory systems should also be examined in order to assess the generalizability of the findings. Second, in the present study we only assessed learning outcomes a few minutes or less after encoding, for both declarative and procedural memory. However, given that math is learned over much longer periods, and that consolidation in the memory systems may depend on at least partly distinct neurocognitive mechanisms from those underlying initial learning [32,61], investigating the relationship between math and longer-term retention in the memory systems is clearly warranted. Third, and importantly, this study used a general neuropsychological standardized test [36] to assess math ability. Although our positive results linking declarative memory to the measures from this test indeed suggest a reliance of math abilities on this memory system, they do not reveal just *which* aspects of math probed in the standardized test depend on this system–nor whether other aspects of math may be learned in either memory system. Future studies should focus on specific areas of arithmetic and other aspects of math to advance our understanding of this issue. Fourth, although here we controlled for the basic demographic variables of sex and age, future studies should also address other potentially confounding factors, such as socioeconomic status, IQ, or cognitive abilities such as working memory–even though the potential influence of this last factor on our declarative learning measure was minimized by virtue of our relatively process-pure task design (see Methods). Fifth, although a clear strength of this study is its longitudinal design, moreover with a not-insubstantial number of participants, a larger study, moreover with a broader grade-range, would be desirable. In particular, a larger sample of participants could further increase the generalizability of the results and reduce the possibility of model overfitting. Sixth, although the results appear to be robust, in that the analyses provided robust, bootstrapped estimates and corrected confidence intervals of model parameters, and moreover survived three sensitivity analyses, other robustness checks could be added in future studies, such as analyses of random halves of the sample (which did not yield convergence in this study) or a leave-one-out approach (which was not examined due to computational demands). Seventh, our SEM that included a subject-level random intercept for each longitudinal measure to control for stable, between-person differences [62], resulted in null effects, primarily due low power. As such, we cannot rule out that our significant indirect effects may be due to differences between subjects, rather than effects between measures across time. We suggest that future work use a larger sample size to facilitate the inclusion of such random intercepts, in order to better test the causal relationship between memory and math ability. Eighth, this purely behavioral study should be followed up with neuroimaging and other approaches that can assess the neural predictions of the DP model of math [11]. Indeed, we hope that the present study will provide an empirical foundation for further research that examines the range of predictions of this framework, and further specifies the model. Finally, whereas this paper focused on typically developing individuals, future work should test the relations between the memory systems and math abilities in developmental math disability, in particular examining the predictions of the Procedural Deficit Hypothesis (PDH), which is built upon the DP model of math [11].

### Conclusions, and implications for math learning and interventions in struggling students

The results of this study highlight the role of declarative memory in the development of children’s mathematical abilities, consistent with the DP model of math. The study suggests new areas of research investigating whether, to what extent, when, and how the declarative and/or procedural memory systems scaffold the learning of math. From a more applied perspective, the findings suggest both diagnostic and intervention strategies for students struggling with math, including the possibility that behavioral and other techniques that have independently been shown to improve learning in the memory systems [63,64] may also be employed to improve learning in the critical domain of math.

## Supporting information

S1 Text Supporting information. S1 Table. *Indirect Effects of No-Covariate Model*. S2 Table. *Indirect Effects of Restricted Sample Model with Procedural Memory*. S3 Table. *Indirect Effects of Restricted Sample Model without Procedural Memory*. S4 Table. *Fitted Series of Mixed-Effect Models Testing Declarative and Procedural Memory’s Relationships with Mathematical Ability Across Grades 2 to 4*. S5 Table. *Correlation-Based SEM Parameters*, *with and without Longitudinal Random Intercepts*. S6 Table. *Correlation-Based SEM Indirect Effects*, *with and without Longitudinal Random Intercepts*. S7 Table. *Average Model Coefficients and Standard Errors in Monte Carlo Power Analyses*. S8 Table. *Correlation-Based SEM Indirect Effects*, *with and without Longitudinal Random Intercepts*. S1 Fig. SEM without covariates. S2 Fig. Sample-restricted SEM with procedural memory. S3 Fig. Sample-restricted SEM without procedural memory. S4 Fig. SEM with autoregressive paths. Appendix 1 Mplus Code for doing correlation matrix-based SEM with longitudinal random intercepts. Appendix 2 R Studio Code for performing the Monte Carlo Method for Assessing Mediation (MCMAM) from the correlation-based model parameters. Appendix 3 Mplus Code for Monte Carlo power simulations of the longitudinal random intercept SEM using different number of subjects, missing data, and indirect effect size.
(DOCX)
