# Correction: Declarative memory supports children’s math skills: A longitudinal study

**DOI:** 10.1371/journal.pone.0358526

**Published:** 2026-09-15

**Authors:** The PLOS One Staff

## Notice of Republication

This article was republished on September 2, 2026, to remove Supporting Information files that were incorrectly included in the originally published article. The publisher apologizes for this error.
