# Supplementary material for: Declarative memory supports children’s math skills: A longitudinal study
Source: PLoS One. 2024 Jul 25;19(7):e0304211. doi: 10.1371/journal.pone.0304211 (PMC11271893; doi:10.1371/journal.pone.0304211)
Supplement: S1 File — (PDF) [file pone.0304211.s003.pdf]

Vanderbilt University
Institutional Review Board
504 Oxford House Nashville, Tennessee 37232-4315
(615) 322-2918 Fax: (615) 343-2648
www.mc.vanderbilt.edu/irb

June 8, 2016

Laurie Cutting, Ph.D.
Kennedy Center
Peabody College Box 328 37203-5721

RE: IRB# 160931 "The Neurocognition of Procedural and Declarative Memory in Dyslexia and S-RCD" (NIH)

Dear Laurie Cutting, Ph.D.:

A sub-committee of the Institutional Review Board reviewed the research application identified above. The sub-committee determined the study poses minimal risk to participants, and the application is approved under 45 CFR 46.110 (F)(4), (6) and (7).

Documentation of informed consent is waived in accordance with 45 CFR 46.117 (c)(2) for the phone screening.

The Consent Form(s) have been stamped with the approval and expiration date and this copy should be used when obtaining the participant's signature. Federal regulations require that the original copy of the participant's consent be maintained in the principal investigator's files and that a copy be given to the subject at the time of consent. An additional record (i.e., case report form, medical record, database, etc.) of the consent process should also be maintained in a separate location for documentation purposes.

As the Principal Investigator, you are responsible for the accurate documentation, investigation and follow-up of all possible study-related adverse events and unanticipated problems involving risks to participants or others. The IRB Adverse Event reporting policy III.G is located on the IRB website at http://www.mc.vanderbilt.edu/irb/.

If this trial requires registration as a clinical trial, accrual cannot begin until this study has been registered at clinicaltrials.gov and a National Clinical Trial Number (NCT) provided. Please provide the NCT# to the IRB as soon as it is obtained. If an approval is required from an additional source other than the Vanderbilt IRB, this must be obtained prior to study initiation. These approvals may include, but are not limited to CRC, SRC, IND, IDE.

Please note that approval is for a 12-month period. Any changes to the research study must be presented to the IRB for approval prior to implementation.

DATE OF IRB APPROVAL: June 8, 2016   DATE OF IRB EXPIRATION: June 7, 2017

Sincerely,

David G. Schlundt, Ph.D., Chair
Institutional Review Board
Behavioral Sciences Committee
DGS/jga
Electronic Signature: David G Schlundt/VUMC/Vanderbilt : (796F1AC8B041640E8B9C1882CCA9AE5D)
Signed On: 06/08/2016 01:21:01 PM CDT
