# Supplementary material for: Declarative memory supports children’s math skills: A longitudinal study
Source: PLoS One. 2024 Jul 25;19(7):e0304211. doi: 10.1371/journal.pone.0304211 (PMC11271893; doi:10.1371/journal.pone.0304211)
Supplement: S3 File — (PDF) [file pone.0304211.s005.pdf]

VANDERBILT

RE: IRB #160931 "The Neurocognition of Procedural and Declarative Memory in Dyslexia and S-RCD"

Dear Laurie E Cutting, Ph.D.:

A sub-committee of the Institutional Review Board reviewed the Application for Continuing Review for the research study identified above. The sub-committee determined the study poses Minimal Risk to participants. This study meets 45 CFR 46.110 (F) category (4),(6),and(7) for Expedited Review.

The Consent Form(s) have been stamped with the approval and expiration date and this copy should be used when obtaining the participant's signature. Federal regulations require the original copy of the participant's consent be maintained in the principal investigator's files and that a copy be given to the participant at the time of consent. An additional record (i.e., case report form, medical record, database, etc.) of the consent process should also be maintained in a separate location for documentation purposes.

Please note the requirement for annual VU IRB Human Subjects Training is not current or will soon expire for some key study personnel (KSP) associated with this study. It is the Principal Investigator’s responsibility to ensure that all KSP have met the annual training requirement (see IRB Procedure VI.B.1). Please log in to DISCOVR-E, select the KSP tab for the approved study and review the IRB training status to identify those who need to renew training. Those individuals may then access the IRB Education page for education options (in-person and online) relative to the IRB Basics course or refresher modules https://wp0.vanderbilt.edu/irb/vhrppeducation/ .

As the Principal Investigator, you are responsible for the accurate documentation, investigation and follow-up of all possible study-related adverse events and unanticipated problems involving risks to participants or others. The IRB Adverse Event reporting policy III.G is located on the IRB website at http://www.mc.vanderbilt.edu/irb/.

Please note that approval is for a 12-month period. Any changes to the research study must be presented to the IRB for approval prior to implementation.

DATE OF IRB APPROVAL: 4/23/2018

DATE OF IRB EXPIRATION: 4/22/2019

Sincerely,

David G Schlundt PhD, Chair
Institutional Review Board
Behavioral Sciences Committee

Electronic Signature: David G Schlundt/VUMC/Vanderbilt : (e83724dec6d6aeb6f0121ec74121b2e2)
Signed On: 04/24/2018 7:08:51 AM CDT

1313 21st Ave., South, Suite 504
Nashville, TN 37232
www.vanderbilt.edu/irb
