# Supplementary material for: Declarative memory supports children’s math skills: A longitudinal study
Source: PLoS One. 2024 Jul 25;19(7):e0304211. doi: 10.1371/journal.pone.0304211 (PMC11271893; doi:10.1371/journal.pone.0304211)
Supplement: S4 File — (DOCX) [file pone.0304211.s006.docx]

**Supporting Information**

This Supporting (Supplementary) Information provides additional details related to our analyses, further explains our reasoning for certain decisions in our analyses, presents sensitivity analyses for our structural equation models (SEMs), and details our attempts to include a longitudinal random intercept in our SEMs and how it could potentially impact our results. Including this information in the paper would substantially increase the length of the Methods and Results sections, and would distract from the primary goals of connecting our analyses to our research questions.

*Software*

Data management and mixed-effects modeling were handled in STATA 15.1^1^. Structural Equation Models were run in MPlus 8.4^2^.

*Data for the Mixed-Effects Models*

The mixed-effects models used a long data format, in which 261 unique scores for each child in each grade constituted the separate rows. These observations came from 109 children. Level one of our model represented within-child variability of scores (math ability, declarative memory, procedural memory) across grades 2, 3, and 4, which allowed us to test how math ability varied within-children across grades and according to memory scores across these grades. Level two of the mixed-effects models accounted for multiple observations over time belonging to a single child, allowing estimation of effects that vary only between children. By representing the nesting of scores within children, the model adjusted standard errors and covariances of variables based on the non-independence of observations.

*Model Comparisons*

Akaike Information Criterion (AIC) and Bayesian Information Criterion (BIC) are relative metrics of fit used to compare nested models. A model with additional parameters (e.g., more variables in a regression, or the addition of random intercepts) shows better fit to the data if it has lower information criterion values compared to a simpler nested model without those parameters. Information criterion weighs the increase in model fit to a built-in parsimony penalty for having additional parameters, such that the value of additional variables must outweigh their cost to complexity^1^. While direct, falsifiable tests to determine improvements in model fit exist (e.g. the Log-Likelihood Test), they are either inapplicable to mixed-effects models or are invalid with robust standard errors. Because of the non-normality of our data and our use of robust standard errors, we relied on AIC and BIC to conduct mixed-effects model comparisons.

For comparisons of SEMs, we used the Satorra-Bentler scaled chi-square difference test^2,3^. This is a non-normal-robust test which assesses if a less-restricted baseline model (i.e., one with more parameters) shows better-fit than a more-restricted nested model (i.e., one with constraints or fewer parameters). A significant test statistic indicates that the more complex model is a better fit for the data. The Satorra-Bentler is valid even when the compared models use robust standard errors.

*Bootstrapping and Indirect Effects for the SEMs*

Estimating distributions of indirect effect coefficients can be problematic because a distribution of multiplicative products will be non-normal and imbalanced^4,5^. One method that is commonly employed to address this issue is to build an empirical distribution of indirect effect products obtained through bootstrap resampling. Such a distribution has known properties that allow the confidence intervals (*CI*s) to be determined for the possible indirect effect values^4,6^. Though the bootstrapped distribution can still be biased and diverge from a true population mean, bias-corrected *CI*s for the indirect effect estimates, as were used in our analyses, can address this problem^5^.

*Small Sample Concerns for the SEMs*

The sample size of 109 participants, many of which had missing data (i.e., missing math or memory values in one or more grades; see next subsection for specifics), may be a cause for concern for our SEM results. To this end we took measures to maximize the validity of our analyses with the data that we had, in particular bootstrapping and bias-corrected *CI*s (see just above)^5,6^. Our models indeed converged, fulfilled solution propriety (i.e., the model converged without impossible parameter estimates^7^, and were an excellent fit for our data. Further, the SEM sensitivity analyses (see below) suggest that the pattern of findings are robust, and thus small sample size or missing data are unlikely to have led to spurious results.

Other work looking at SEM integrity under different conditions has suggested relatively small sample size requirements, including below 100 participants, to obtain satisfactory models with latent variables, though there is substantial variation in model quality based on missing data and effect sizes^7,8^. It is somewhat unclear how this work directly translates to our simpler SEM regressions using observed (rather than latent) variables. Note also that while bootstrapping and bias correction are preferrable to other methods in addressing the potential for inflated type I errors for indirect effects obtained from small samples^6^, even with these techniques there still may be cause for concern for such errors^9^. Such lingering concerns prompted us to report more conservative 99% *CI*s for the indirect effects, as well as to conduct sensitivity analyses to examine the robustness of the effects under different conditions (see below).

*Missing Data Concerns for the SEMs*

Missing data resulted from a combination of participant attrition, participants joining the study after the first time point, and non-responses. The total data profile at the child level across the grades was as follows: 24 children had data from all three measures (math ability, declarative memory, procedural memory) in all three grades, 8 had data for all three measures in grade 2 only, 10 in grades 2 and 3 only, 3 in grades 2 and 4 only, 13 in grade 3 only, 34 in grades 3 and 4 only, 5 in grade 4 only, and 12 had some data missing for one or more of these measures in all three grades. Procedural memory variables had the highest amount of missing data (56 percent missing in grade 2, 24 percent in grade 3, and 39 percent in grade 4). Despite this, procedural memory was still valuable in our models since it helped statistically isolate the unique effects of declarative memory and improved model fit.

For the SEMs, this missing data issue would have drastically affected sample size if we had used standard case-wise deletion. To use all data from all 109 children, we therefore utilized the Full Information Maximum Likelihood (FIML) technique, a model-based imputation method. FIML allowed us use all available information for parameter and standard error estimates, as well as to have consistent sample sizes for more valid model comparisons. The only model specification needed for FIML is that all continuous exogenous variables covary, which for our models included declarative and procedural memory in grade 2, along with age in each grade. See Enders & Bandalos^10^ for further explanation of FIML, and for evidence from a simulation study which supported its use compared to other missing data methods.

*SEM Sensitivity Analyses*

Despite the concerns discussed above regarding sample size and missing data, the key SEM results regarding indirect effects were robust, in that sensitivity (alternate) analyses revealed that declarative memory abilities in earlier grades always significantly predicted later math abilities. That is, declarative memory in grade 2 consistently predicted math ability in both grades 3 and 4, and declarative memory in grade 3 predict math ability in grade 4. This pattern held not only with 95% *CI*s, but even with 99% *CI*s.

We performed three SEM sensitivity analyses. The first model included no covariates (that is, both the sex and age covariates were removed). The fit for the final model reported in the paper, that is, the bootstrapped model with covariates (*CFI* = .97; *RMSEA* =.03), was better than for the sensitivity model without covariates (*CFI =* .96; *RMSEA* = .06). Nevertheless, the significance pattern of all indirect effects remained the same as in the final model (with both 95% and 99% *CI*s), as did all direct effects except for one (declarative memory in grade 4 predicting math ability in the same grade, which became non-significant [*b* = 0.18, *SE* = 0.10, *p* = .07], unlike in the final model [*b* = 0.19, *SE* = 0.09, *p* = .04]). See Supplementary Table 1 and Supplementary Figure 1.

In the second sensitivity analysis, we included only children who had complete data for both declarative memory and math ability, that is, children for whom we had both of these measures from all three grades. This left us with 61 out of the 109 participants. This analysis was designed to address potential issues regarding missing data (see above). Note that further constraining the sample to only those participants who additionally had procedural memory measures from all three grades would leave only 24 children in the sample, which is insufficient for model estimation. We performed two sensitivity analyses on this restricted sample of 61 participants: one that included procedural memory (using FIML to retain cases) and another that removed procedural memory (given that data was missing for this task from a large proportion of the participants). To minimize differences with the final model, we retained both age and sex as covariates in both of these sensitivity analyses.

The restricted sample model that retained procedural memory had much worse fit (*CFI* = .80; *RMSEA* = .11) than the final model. Nevertheless, as in the first sensitivity analysis, this model yielded the same significant direct effects for declarative memory and math ability except for one (again, declarative memory in grade 4 predicting math ability in the same grade became non-significant [*b* = 0.14, *SE* = 0.11, *p* = .21]). Moreover, the indirect effects were broadly similar to the final model (and the first sensitivity analysis). With a *CI* of 95%, all indirect effects were significant except those that were already not significant with a *CI* of 99% in the final model (both effects involving DM 4 → Math 4, likely because of the unreliability of that direct effect). Moreover, two additional effects became non-significant at *CI* 99% (both included DM 2 → Math 2, possibly due to a higher standard error for that direct effect [*SE* = 0.10] in this model than in the final model [*SE* = 0.06]). Crucially, in this restricted sample model declarative memory abilities in earlier grades always significantly predicted later math abilities, even if certain specific indirect paths dropped out. Thus, with both *CI* 95% and *CI* 99%, declarative memory in grade 2 still predicted math ability in grades 3 and 4 through other indirect pathways, and likewise declarative memory in grade 3 still predicted math ability in grade 4. See Supplementary Table 2 and Supplementary Figure 2.

The third sensitivity analysis, with the same restricted sample of 61 participants, but without the inclusion of procedural memory, also had lower fit than the final model (*CFI* = .87; *RMSEA* = .11). Nevertheless, this analysis yielded the same significant direct effects as the final model except for one (declarative memory in grade 4 predicting math ability in the same grade was again non-significant [*b* = 0.07, *SE* = 0.10, *p* = .45]). Moreover, the indirect effects again showed a similar pattern as the final model. In this case the only difference with the final model was that in this sensitivity analysis those indirect effects that were not significant with a *CI* of 99% in the final model were also not significant with a *CI* of 95% in this model (both effects involving DM 4 → Math 4). See Supplementary Table 3 and Supplementary Figure 3.

In sum, all three sensitivity analyses yielded the same pattern of significant direct effects between declarative memory and math ability except for the effect of declarative memory on math ability within grade 4. Moreover, although certain specific indirect paths dropped out in the second and/or third sensitivity analysis (mainly the two effects involving DM 4 → Math 4, likely because of the unreliability of that direct effect), in all three sensitivity analyses declarative memory abilities in earlier grades significantly predicted (at both 95% and 99% confidence intervals) math abilities in later grades via one or more indirect effects, including from grades 2 to 4, grades 2 to 3, and grades 3 to 4.

*Implementing a Longitudinal Random Intercept Model*

Our original analysis for the longitudinal relationships between declarative memory, procedural memory, and math ability from 2^nd^ to 4^th^ grade was intended to be a Random Intercept Cross-Lagged Panel Model (RICLPM^11^), which estimates the direct predictive relationship of different variables between timepoints (e.g., DM 2 → Math 3) while including latent factors to control for stable differences within a measure across timepoints. This model design helps to separate the variation between timepoints that is shared across subjects from the variation *within*a subject, such that we can better test if a subject's increase in DM or PM from their baseline at an earlier timepoint predicts their own increases in Math at a later timepoint. Without this random intercept, our model addresses whether students with higher DM or PM have higher math performance at the next timepoint compared to students with lower DM or PM.

Implementing the RICLPM proved difficult because of our sample size and missing data. First, the relationships between memory and math between timepoints would often not reach statistical significance, while predictive relationships within the same timepoint would reach significance (e.g., DM 3 → Math 3), even when controlling for previous math ability. This was the reason we utilized indirect effects, which, while not allowing us to make as strong of a causal claim with the clear temporal precedence of memory before math ability, would at least allow us to test if math performance in a given grade could be significantly linked to either DM or PM in the previous grade. We will refer to this model variant as the longitudinal random intercept SEM.

The second barrier to implementing a random intercept in our SEM was that it would result in model nonconvergence due to estimation problems of the standard errors, likely due to estimating three random intercepts (one each for DM, PM, and Math), a relatively small sample, and missing data. This failed estimation occurred regardless of whether PM was included or not, or whether the data was restricted to those with complete data on the model variables.

The only longitudinal random intercept SEM that succeeded in estimation was one that used the correlation matrix of the variables, though this method assumed complete data for all 109 cases and did not achieve convergence when sex and age were added as covariates. We ran an indirect effects model with the random intercept “deactivated” by setting the variance and covariance of the random intercepts to zero^12^, and compared the results to a model with the random intercept “activated” to see how its inclusion could impact our results. Due to the inability to use bootstrapping methods to estimate indirect effects with a correlation matrix dataset, another required change was to estimate the indirect effects with the Monte Carlo Method for Assessing Mediation (MCMAM^13^), which utilizes the coefficient estimates, variances, and covariances to simulate a distribution of one-mediator indirect effects (such that a two mediator effect, like DM 2 → DM 3 → Math 3 → Math 4 could not be readily calculated). If the 95% confidence interval of these indirect effects did not include 0, then the indirect effect was considered significant. See Appendix 1 for the Mplus code used to perform the correlation-based random-intercept models, and Appendix 2 for the R Studio code used to estimate the indirect effects of the random intercept models using MCMAM.

In the model with the inactive random intercept (CFI = 0.90, RMSEA = .12), all estimated one-mediator indirect effects were shown to be different from zero, similar to our original indirect effect models. These results would suggest that students with higher DM in a given grade are likely to show higher math ability in the subsequent grade. Comparatively, in the model that included the random intercept for DM, PM, and math (CFI = 0.95, RMSEA = .11), none of these indirect effects were sufficiently different from zero. The null indirect effects were likely due to a decrease in the size of autoregressive effects and between-variable predictive effects, alongside an increase in the coefficient standard errors. The null results of the longitudinal random intercept SEM would suggest that individual increases in DM or PM are not related to their math abilities at later times, accounting for stable sources of variation that could exist between subjects (e.g., age-related development). See Supplementary Table 5 for the model parameters, and Supplementary Table 6 for the indirect effect estimates.

However, given our estimation issues with implementing the longitudinal random intercept SEM, we ran several Monte Carlo simulations^14,15^ to ascertain the power of this model to successfully estimate a truly non-zero indirect effect given our sample size and extent of missing data, and to help future research select a sufficient sample size for similar questions. We only included DM and Math ability in this simulation in the interest of simplicity and computation time. For the population parameters of the simulation to test the power of our own data, we specified having 109 subjects to match our actual sample, fixed the population means and variances of the DM and Math variables to those we observed in our data at the appropriate timepoints, and incorporated the observed missing data proportions for each variable (Math 2 = 15.60% missing, Math 3 = 18.35%, Math 4 = 26.61%, DM 2 = 19.27%, DM 3 = 20.18%, DM 4 = 27.52%) for the generated datasets. We assigned all one-mediator indirect effects (e.g., DM 2 → DM 3 → Math 2) to have an indirect effect coefficient of .15 in the longitudinal random intercept SEM with each autoregression coefficient (e.g., Math 2 → Math 3) being set to .50, and fixing each between-variable effect to .30 (.50 * .30 = .15). The Monte Carlo simulation estimated all indirect effects using the Bayes estimator, 1,000 total iterations^[[1]](#footnote-1)^, and two Markov Chain Monte Carlo chains with an iteration length of 100,000 per chain to reach the Gelman-Rubin .05 convergence criterion. This simulation was performed twice to see the consistency of results. See Appendix 3 for the Mplus code used to perform these power analyses.

In both simulations, 429 and 438 model replications converged successfully, which highlights general estimation issues that we experienced with including a longitudinal random intercept in our own data. The simulations had the most difficulty calculating coefficients for declarative memory in grade 2, resulting in standard error estimates that were too large to print in the output. The most stable estimated indirect effect, DM 3 → DM 4 → Math 4, had an average estimate across models of .11, and it only had a 95% confidence interval which did not contain zero in 27.2%-27.5% of successful replications, meaning at most there was only about 28% power to find a significant indirect effect of .15 given our 109 subjects and missing data.

We also performed a pair of simulations under three other model conditions: 1) 109 subjects, no missing data, and a true indirect effect of .15; 2) 200 subjects, no missing data, and a true indirect effect of .15; and 3) 200 subjects, no missing data, and a true indirect effect of .10 (by reducing autoregressive coefficients to .40, and cross-variable coefficients to .25). In the first additional simulation set (109 subjects with no missing data), more replications converged (629-664), but with similar instability in the estimates for DM 2. The DM 3 → DM 4 → Math 4 had an average estimate of .12 and still had the highest power, but only at 45.6%-48.2%. In the second additional simulation set (200 subjects and no missing data), many more replications were completed (903-928) and the standard deviation estimates for DM2 were small enough to be printed. The strongest indirect effect was still DM 3 → DM 4 → Math 4, with an average indirect effect of .12, and the power to calculate it was 78.9% to 81.3%. In the final simulation set (200 subjects with a decreased indirect effect of .10), 948 replications were completed in both simulations. The DM 3 → DM 4 → Math 4 indirect effect had an average estimate of .08, and the power was only 53.6% to 55.1% to find a non-zero effect. See Supplementary Table 7 and 8 for the estimated model coefficients and indirect effects respectively.

Given the general estimation problems in implementing random intercepts in our SEM models, and the low power to find an indirect effect in our Monte Carlo simulation, we cannot determine whether or not there is truly a null effect in relating DM or PM in a given grade to Math ability in a later grade when accounting for stable difference between subjects. For future studies we advocate for the use of a random intercept in any longitudinal model for relating memory to math ability, since it can provide stronger causal evidence by addressing if variation in memory at the subject level can predict a subject’s math performance in later grades.

*Supplementary Table 1*

| Outcome | Indirect Pathway | 95% Bias-Corrected Confidence Interval | 99% Bias-Corrected Confidence Interval |
| --- | --- | --- | --- |
| Math Ability 3 |  |  |  |
|  | DM 2 → DM 3 → Math 3 | [0.06, 0.29] | [0.03, 0.34] |
|  | DM 2 → Math 2 → Math 3 | [0.05, 0.24] | [0.02, 0.28] |
| Math Ability 4 |  |  |  |
|  | DM 3 → DM 4 → Math 4 | [0.01, 0.18] | [-0.02, 0.22] |
|  | DM 3 → Math 3 → Math 4 | [0.07, 0.35] | [0.03, 0.41] |
|  | DM 2 → DM 3 → DM 4 → Math 4 | [0.00, 0.12] ^a^ | [-0.01, 0.14] |
|  | DM 2 → DM 3 → Math 3 → Math 4 | [0.04, 0.23] | [0.02, 0.28] |
|  | DM 2 → Math 2 → Math 3 → Math 4 | [0.03, 0.20] | [0.01, 0.23] |

*Indirect Effects of No-Covariate Model*

*Note.* Any confidence interval that did not contain zero was considered significant. Parameter estimates were taken from a bootstrapped model with 10,000 resamples. DM refers to Declarative Memory Scores and Math to Mathematical ability scores. The number at the end of the variable name refers to grade 2, 3, or 4. See Supplementary Figure 1 for a visual representation of the direct effects of this model.

^a^ The lower bound of confidence interval was slightly above 0; thus this effect was significant.

*Supplementary Table 2*

| Outcome | Indirect Pathway | 95% Bias-Corrected Confidence Interval | 99% Bias-Corrected Confidence Interval |
| --- | --- | --- | --- |
| Math Ability 3 |  |  |  |
|  | DM 2 → DM 3 → Math 3 | [0.05, 0.34] | [0.02, 0.41] |
|  | DM 2 → Math 2 → Math 3 | [0.02, 0.29] | [-0.01, 0.36] |
| Math Ability 4 |  |  |  |
|  | DM 3 → DM 4 → Math 4 | [-0.01, 0.14] | [-0.03, 0.18] |
|  | DM 3 → Math 3 → Math 4 | [0.06, 0.42] | [0.01, 0.50] |
|  | DM 2 → DM 3 → DM 4 → Math 4 | [0.00, 0.09] | [-0.02, 0.12] |
|  | DM 2 → DM 3 → Math 3 → Math 4 | [0.04, 0.28] | [0.01, 0.34] |
|  | DM 2 → Math 2 → Math 3 → Math 4 | [0.02, 0.26] | [-0.01, 0.32] |

*Indirect Effects of Restricted Sample Model with Procedural Memory*

*Note.* See Note to Supplementary Table 1 for details. See Supplementary Figure 2 for a visual representation of the direct effects of this model.

*Supplementary Table 3*

| Outcome | Indirect Pathway | 95% Bias-Corrected Confidence Interval | 99% Bias-Corrected Confidence Interval |
| --- | --- | --- | --- |
| Math Ability 3 |  |  |  |
|  | DM 2 → DM 3 → Math 3 | [0.05, 0.32] | [0.02, 0.39] |
|  | DM 2 → Math 2 → Math 3 | [0.05, 0.28] | [0.02, 0.33] |
| Math Ability 4 |  |  |  |
|  | DM 3 → DM 4 → Math 4 | [-0.03, 0.11] | [-0.06, 0.15] |
|  | DM 3 → Math 3 → Math 4 | [0.07, 0.44] | [0.02, 0.52] |
|  | DM 2 → DM 3 → DM 4 → Math 4 | [-0.02, 0.08] | [-0.04, 0.10] |
|  | DM 2 → DM 3 → Math 3 → Math 4 | [0.05, 0.29] | [0.02, 0.36] |
|  | DM 2 → Math 2 → Math 3 → Math 4 | [0.03, 0.26] | [0.00, 0.31] ^a^ |

*Indirect Effects of Restricted Sample Model without Procedural Memory*

*Note.* See Note to Supplementary Table 1 for details. See Supplementary Figure 3 for a visual representation of the direct effects of this model.

^a^ The lower bound of confidence interval was slightly above 0; thus this effect was significant.

*Supplementary Table 4*

| Predictors *b* | Models Predicting Math Ability | | | | | | | | | | |
| --- | --- | --- | --- | --- | --- | --- | --- | --- | --- | --- | --- |
|  | Empty | Covariate | | Memory Prediction | | Memory x Grade | | Memory x Age | | Memory x Male | |
| DM | - | | - | | 0.20*** | | 0.17* | | -0.39 | | 0.20** |
| DM x Grade 2 | - | | - | | - | | -0.07 | | - | | - |
| DM x Grade 4 | - | | - | | - | | 0.12 | | - | | - |
| DM x Age | - | | - | | - | | - | | 0.06 | | - |
| DM x Male | - | | - | | - | | - | | - | | -0.01 |
| PM | - | | - | | -0.04 | | -0.05 | | -0.04 | | -0.04 |
| Grade 2 | - | | -0.17*** | | -0.16** | | -0.13 | | -0.16** | | -0.16*** |
| Grade 4 | - | | 0.13** | | 0.12* | | 0.06 | | 0.12* | | 0.12* |
| Age | - | | 0.01 | | 0.01 | | 0.01 | | -0.02 | | 0.01 |
| Sex (Male) | - | | 0.00 | | 0.00 | | 0.00 | | 0.00 | | 0.01 |
|  |  | |  | |  | |  | |  | |  |
| Intercept | 0.55*** | | 0.45 | | 0.36 | | 0.38 | | 0.64 | | 0.35 |
| Random Intercept (*SD*)^a^ | 0.85* | | 0.13* | | 0.12* | | 0.12* | | 0.12* | | 0.12* |
| Residual *ICC* ^a^ | 0.21* | | 0.68* | | 0.63* | | 0.64* | | 0.63* | | 0.63* |
|  |  | |  | |  | |  | |  | |  |
| AIC | -95.70 | | -213.52 | | -220.84 | | -218.74 | | -219.76 | | -218.85 |
| BIC | -85.92 | | -194.71 | | -191.52 | | -182.91 | | -187.18 | | -186.27 |

*Fitted Series of Mixed-Effect Models Testing Declarative and Procedural Memory’s Relationships with Mathematical Ability Across Grades 2 to 4.*

*Note*. ** p* < .05, ** *p* < .01, *** *p* < .001. DM refers to declarative memory, PM to procedural memory, SD to standard deviation, ICC to Intraclass Correlation, AIC to Akaike Information Criterion, and BIC to Bayesian Information Criterion. The model includes 192 student-grade observations, for 97 subjects. Random Intercepts were estimated between subjects. Samples were kept consistent between models using case wise deletion. The random intercept accounts for observations being nested in children. We used robust standard errors to account for non-normality. Interactions with PM were not tested because PM did now show a significant main effect. The intercept encompasses female children, as well as children in grade 3. The Memory Prediction model (third model shown above) had the best fit; it shows that higher declarative memory predicts higher math achievement across grade 2 to 4; see Results.

^a^ Significant values have a 95% confidence interval that does not include 0.

*Supplementary Table 5*

*Correlation-Based SEM Parameters, with and without Longitudinal Random Intercepts*

|  |  | Inactive RI | | Active RI | |
| --- | --- | --- | --- | --- | --- |
| Outcome | Predictor | b | S.E. | b | S.E. |
|  |  |  |  |  |  |
| Math 2 | DM 2 | 0.29** | 0.09 | 0.02 | 0.15 |
|  | PM 2 | -0.07 | 0.09 | 0.06 | 0.09 |
|  |  |  |  |  |  |
| Math 3 | Math 2 | 0.57*** | 0.08 | 0.01 | 0.24 |
|  | DM 3 | 0.24* | 0.09 | 0.12 | 0.11 |
|  | DM 2 | -0.04 | 0.10 | -0.07 | 0.15 |
|  | PM 3 | 0.00 | 0.07 | 0.03 | 0.08 |
|  |  |  |  |  |  |
| Math 4 | Math 3 | 0.67*** | 0.07 | 0.31^+^ | 0.17 |
|  | DM 4 | 0.22** | 0.07 | 0.22* | 0.11 |
|  | DM 3 | -0.01 | 0.08 | 0.04 | 0.10 |
|  | DM 2 | 0.01 | 0.08 | -0.02 | 0.12 |
|  | PM 4 | -0.12^+^ | 0.06 | -0.11 | 0.07 |
|  |  |  |  |  |  |
| DM 3 | DM 2 | 0.64*** | 0.07 | 0.41** | 0.15 |
|  |  |  |  |  |  |
| DM 4 | DM 3 | 0.40*** | 0.08 | 0.06 | 0.21 |
|  |  |  |  |  |  |
| PM 3 | PM 2 | 0.32*** | 0.09 | 0.21 | 0.14 |
|  |  |  |  |  |  |
| PM 4 | PM 2 | 0.12 | 0.09 | -0.01 | 0.15 |

*Note. * p* < .05, ** *p* < .01, *** *p* < .001. Inactive RI model fit: *CFI* = .90*, RMSEA*= .12*, AIC* = 2565.10*, n-*adjusted *BIC* = 2552.45, *N* = 109. Active RI model fit: *CFI* = .95*, RMSEA*= .11*, AIC* = 2558.79*, n-*adjusted *BIC* = 2543.33, *N* = 109. DM refers to Declarative Memory scores, PM to Procedural Memory scores, and Math to mathematical ability scores. The number at the end of the variable name refers to grade 2, 3, or 4. Due to estimation difficulties, results were obtained from two models that were estimated using the correlation matrix of variables in our data. The models were designed to incorporate a random intercept for each set of variables across timepoints (e.g., DM 2, DM 3, & DM 4), which helps to isolate sources of stable between-subject variance from that within a subject. The results on the left-side, the “Inactive RI”, negates the random intercept, while the results under “Active RI” fully utilizes it.

*Supplementary Table 6*

*Correlation-Based SEM Indirect Effects, with and without Longitudinal Random Intercepts*

|  | Inactive RI | | Active RI | |
| --- | --- | --- | --- | --- |
| Indirect Effects | 95% CI | 99% CI | 95% CI | 99% CI |
| DM 2 → DM 3→ Math 3 | [0.03, 0.28] | [0.00, 0.32] | [-0.04, 0.18] | [-0.07, 0.24] |
| DM 3 → DM 4 → Math 4 | [0.03, 0.16] | [0.01, 0.19] | [-0.11, 0.10] | [-0.17, 0.15] |
| DM 2 → Math 2 → Math 3 | [0.06, 0.28] | [0.03, 0.33] | [-0.07, 0.08] | [-0.12, 0.13] |
| DM 3 → Math 3 → Math 4 | [0.04, 0.29] | [0.00, 0.34] | [-0.03, 0.15] | [-0.06, 0.20] |

*Note.* Any confidence interval that did not contain zero was considered significant. Inactive RI model fit: *CFI* = .90*, RMSEA*= .12*, AIC* = 2565.10*, n-*adjusted *BIC* = 2552.45, *N* = 109. Active RI model fit: *CFI* = .95*, RMSEA*= .11*, AIC* = 2558.79*, n-*adjusted *BIC* = 2543.33, *N* = 109. DM refers to Declarative Memory scores, and Math to mathematical ability scores. The number at the end of the variable name refers to grade 2, 3, or 4. These indirect effects come from the models described in Supplementary Table 5*.* The models were designed to incorporate a random intercept for each set of variables across timepoints (e.g., DM 2, DM 3, & DM 4), which helps to isolate sources of stable between-subject variance from that within a subject. The results on the left-side, the “Inactive RI”, negates the random intercept, while the results under “Active RI” fully utilizes it.

*Supplementary Table 7*

*Average Model Coefficients and Standard Errors in Monte Carlo Power Analyses*

|  |  |  | 109 Subjects, Missing Data, .15 Indirect Effect | | 109 Subjects, No Missing Data, .15 Indirect Effect | | 200 Subjects, No Missing Data, .15 Indirect Effect | | 200 Subjects, No Missing Data, .10 Indirect Effect | |
| --- | --- | --- | --- | --- | --- | --- | --- | --- | --- | --- |
| Outcome | Predictor | Simulation # | Average b | Average S.E. | Average b | Average S.E. | Average b | Average S.E. | Average b | Average S.E. |
| Math 2 | DM 2 | 1 | 56.44 | 19620.55 | 57.38 | 27137.01 | 0.26 | 0.16 | 0.22 | 0.14 |
|  |  | 2 | -227.51 | 13155.58 | 247.11 | 29289.13 | 0.26 | 0.16 | 0.22 | 0.13 |
|  |  |  |  |  |  |  |  |  |  |  |
| Math 3 | Math 2 | 1 | 0.30 | 2.43 | 0.36 | 3.54 | 0.39 | 0.25 | 0.33 | 0.19 |
|  |  | 2 | 0.32 | 0.20 | 0.35 | 0.90 | 0.41 | 0.27 | 0.33 | 0.19 |
|  | DM 3 | 1 | 0.43 | 26617.69 | 0.33 | 14672.51 | 0.31* | 1338.43 | 0.23^+^ | 711.91 |
|  |  | 2 | 0.39 | 26195.91 | 0.34 | 13033.95 | 0.31* | 1636.38 | 0.25^+^ | 813.59 |
|  | DM 2 | 1 | 8440.92 | NA | 125.32 | NA | 0.07 | 1975.20 | 0.02 | 792.46 |
|  |  | 2 | -396.85 | 6396.40 | 52.91 | NA | 0.05 | 2930.13 | 0.03 | 2384.84 |
|  |  |  |  |  |  |  |  |  |  |  |
| Math 4 | Math 3 | 1 | 0.41 | 0.75 | 0.41 | 0.51 | 0.40 | 0.22 | 0.33 | 0.21 |
|  |  | 2 | 0.43 | 0.74 | 0.40 | 0.48 | 0.42 | 0.21 | 0.33 | 0.21 |
|  | DM 4 | 1 | 0.30 | 0.48 | 0.31* | 0.19 | 0.30* | 0.07 | 0.25* | 0.08 |
|  |  | 2 | 0.29 | 0.12 | 0.30* | 0.16 | 0.31* | 0.07 | 0.26* | 0.07 |
|  | DM 3 | 1 | 0.16 | 35982.06 | 0.15 | 18517.08 | 0.09 | 1597.55 | 0.05 | 859.02 |
|  |  | 2 | -0.07 | 36007.29 | 0.15 | 16778.63 | 0.08 | 1962.88 | 0.05 | 1163.53 |
|  | DM 2 | 1 | 9766.01 | NA | 83.11 | NA | 0.08 | 2233.81 | 0.03 | 870.35 |
|  |  | 2 | -621.70 | NA | -30.62 | NA | 0.07 | 3353.78 | 0.04 | 3483.46 |
|  |  |  |  |  |  |  |  |  |  |  |
| DM 3 | DM 2 | 1 | -116.34 | 85816.12 | 1477.92 | 46201.43 | 0.27 | 0.26 | 0.24 | 0.19 |
|  |  | 2 | -256.17 | 81212.95 | 417.10 | 56036.07 | 0.27 | 0.25 | 0.24 | 0.20 |
|  |  |  |  |  |  |  |  |  |  |  |
| DM 4 | DM 3 | 1 | 0.41 | 185.97 | 0.41 | 0.20 | 0.41* | 0.13 | 0.32 | 0.14 |
|  |  | 2 | 0.42 | 3.88 | 0.41 | 0.21 | 0.41^+^ | 0.13 | 0.31 | 0.14 |

*Note. ^+^* 75% to 80% of the confidence intervals for the effect in the simulation did not contain 0, *** Over 80% of the confidence intervals for the effect in the simulation did not contain 0. NA indicates the estimate was too large to be printed or was undefined in the results. DM refers to Declarative Memory scores, and Math to mathematical ability scores. The number at the end of the variable name refers to grade 2, 3, or 4. This table presents the average coefficient size and standard errors of direct effects in four separate Monte Carlo simulations of models where DM predicts Math in later grades (PM was excluded to reduce computational intensity). These simulations also estimated the power to find a non-zero effect based on the percentage of replications where the confidence interval of a given coefficient did not contain zero. All models had longitudinal random intercepts for both DM and Math, the indirect effect of DM predicting Math (e.g., DM 3 -> DM 4 -> Math 4) was fixed at either 0.15 or 0.10, the means and variances of the variables were fixed to what was observed in our data, and each simulation was run twice with 1,000 replications each. The first simulation was designed to match our data with 109 subjects and with missing data in the proportions observed in our data (see Appendix 3 for the missing data proportions), assuming an indirect effect size of 0.15 for all one-mediator indirect effects. The second simulation assumed all data was non-missing with the other conditions remaining the same. The third simulation also assumed no missing data and increased the sample to 200 subjects. The fourth simulation had 200 subjects and no missing data but reduced the magnitude of all tested indirect effects to 0.10. Each effect in the table has two rows, with the first representing the results of the first simulation and the second representing the results of the second simulation for each model set.

*Supplementary Table 8*

*Correlation-Based SEM Indirect Effects, with and without Longitudinal Random Intercepts*

|  |  | 109 Subjects, Missing Data, .15 Indirect Effect | | 109 Subjects, No Missing Data, .15 Indirect Effect | | 200 Subjects, No Missing Data, .15 Indirect Effect | | 200 Subjects, No Missing Data, .10 Indirect Effect | |
| --- | --- | --- | --- | --- | --- | --- | --- | --- | --- |
| Indirect Effects | Simulation # | Average Effect | Average S.E. | Average Effect | Average S.E. | Average Effect | Average S.E. | Average Effect | Average S.E. |
| DM 2 → DM 3→ Math 3 | 1 | -8253.58 | NA | -0.72 | NA | 0.08 | 1975.14 | 0.07 | 792.40 |
|  | 2 | 3.77 | NA | -104.56 | NA | 0.09 | 2930.05 | 0.06 | 2384.78 |
| DM 3 → DM 4 → Math 4 | 1 | 0.11 | 144.74 | 0.12 | 0.19 | 0.12* | 0.05 | 0.08 | 0.05 |
|  | 2 | 0.11 | 1.60 | 0.12 | 0.06 | 0.12^+^ | 0.04 | 0.08 | 0.04 |
| DM 2 → Math 2 → Math 3 | 1 | -27.14 | NA | 60.44 | NA | 0.10 | 0.11 | 0.07 | 0.07 |
|  | 2 | -195.27 | NA | 28.36 | 71207.82 | 0.10 | 0.11 | 0.07 | 0.07 |
| DM 3 → Math 3 → Math 4 | 1 | 0.13 | 25580.77 | 0.11 | 18517.08 | 0.12 | 881.20 | 0.07 | 504.53 |
|  | 2 | 0.13 | 25366.05 | 0.10 | 11360.39 | 0.12 | 1263.10 | 0.07 | 796.11 |

*Note. +* 75% to 80% of the confidence intervals for the effect in the simulation did not contain 0, *** Over 80% of the confidence intervals for the effect in the simulation did not contain 0. NA indicates the estimate was too large to be printed or was undefined in the results. DM refers to Declarative Memory scores, and Math to mathematical ability scores. The number at the end of the variable name refers to grade 2, 3, or 4. This table presents the average coefficient size and standard errors of indirect effects in four separate Monte Carlo simulations of models where DM predicts Math in later grades (PM was excluded to reduce computational intensity). These simulations also estimated the power to find a non-zero indirect effect based on the percentage of replications where the confidence interval of a given indirect effect coefficient did not contain zero. All models had longitudinal random intercepts for both DM and Math, the indirect effect of DM predicting Math (e.g., DM 3 -> DM 4 -> Math 4) was fixed at either 0.15 or 0.10, the means and variances of the variables were fixed to what was observed in our data, and each simulation was run twice with 1,000 replications each. The first simulation was designed to match our data with 109 subjects and with missing data in the proportions observed in our data (see Appendix 3 for the missing data proportions), assuming an indirect effect size of 0.15 for all one-mediator indirect effects. The second simulation assumed all data was non-missing with the other conditions remaining the same. The third simulation also assumed no missing data and increased the sample to 200 subjects. The fourth simulation had 200 subjects and no missing data but reduced the magnitude of all tested indirect effects to 0.10. Each effect in the table has two rows, with the first representing the results of the first simulation and the second representing the results of the second simulation for each model set.


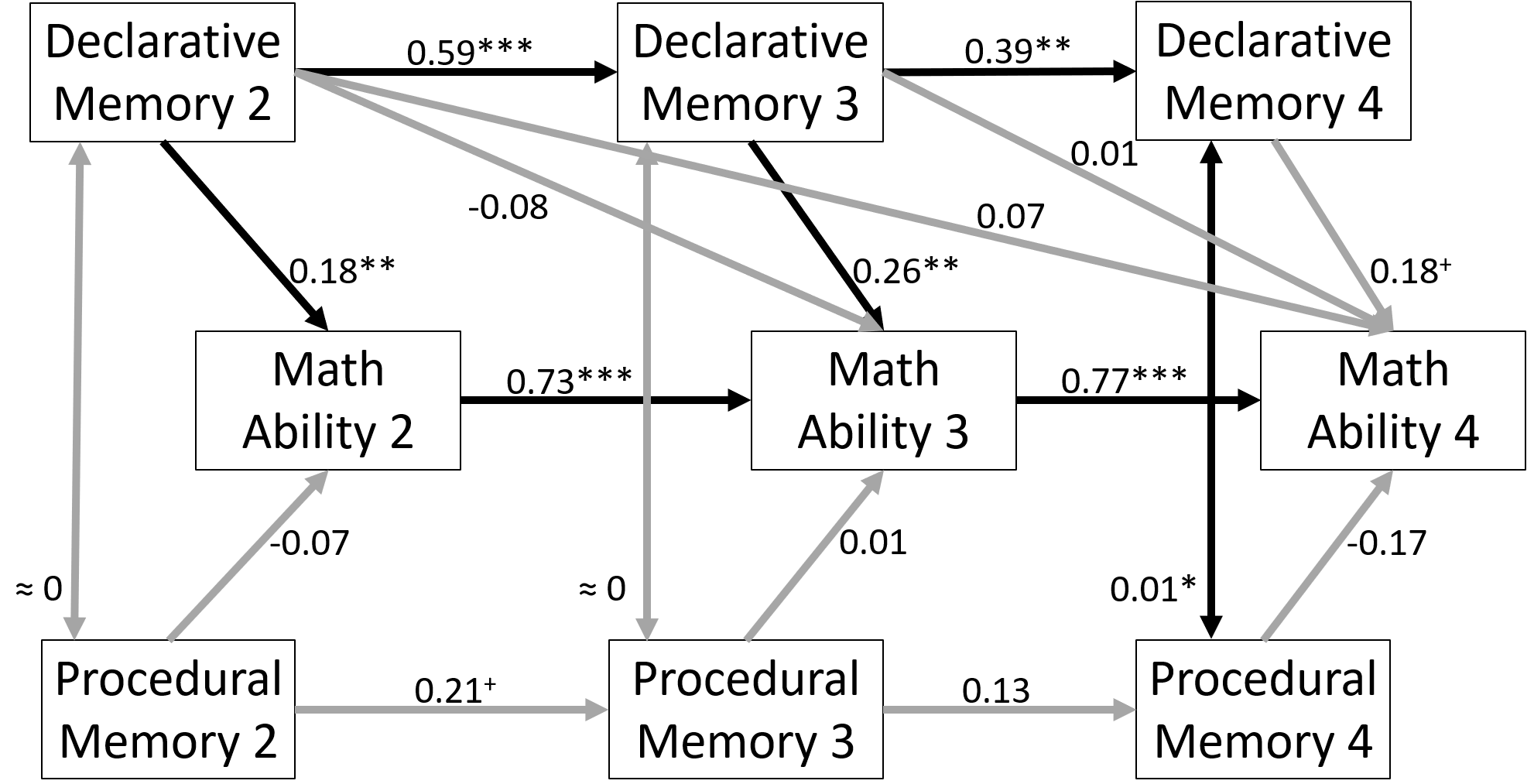


*Supplementary Figure 1.* SEM without covariates. *CFI* = .96*, RMSEA*= .06*, AIC* = -765.94*, n-*adjusted *BIC* = -782.80, *N* = 109. The number at the end of the variable name refers to grade 2, 3, or 4. Lines with single arrows represent regressions, while double-arrow lines (between declarative memory and procedural memory) show covariances. Significant (*p* < .05) paths are in black, while non-significant ones are in gray. Unstandardized coefficients (*b*) are presented adjacent to the path lines. Data for the memory and math ability variables were transformed using Proportion of Maximum Scaling and were person-centered for each grade. *^+^ p* < .10, ** p* < .05*, ** p* < .01*, *** p* < .001.


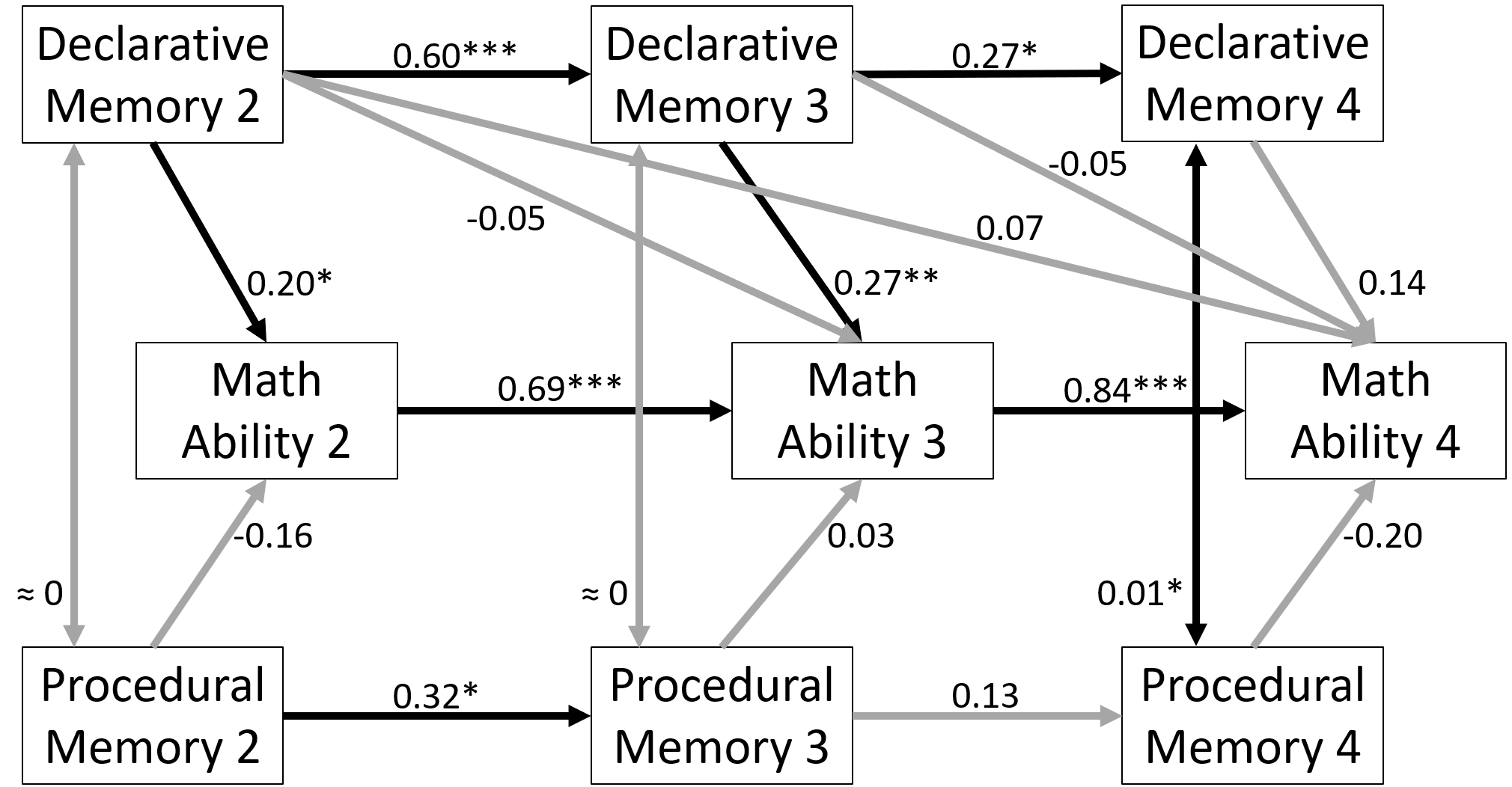


|  |  | Outcome Variable | | |  |
| --- | --- | --- | --- | --- | --- |
| Covariate | Math Ability 2 | | Math Ability 3 | Math Ability 4 | |
| Sex | 0.03 | | 0.02 | -0.03 | |
| Age | 0.05 | | -0.02 | -0.10* | |

*Supplementary Figure 2.* Sample-restricted SEM with procedural memory. *CFI* = .80*, RMSEA*= .11, *AIC* = -753.35*, n-*adjusted *BIC* = -812.35, *N* = 61. Math ability regressions included sex and age in that grade as covariates; these coefficients are presented just below the Figure. Participants were excluded if there was any missing information for declarative memory or math ability in any grade, whereas participants with missing information for procedural memory were retained using FIML. See Note to Supplementary Figure 1 for additional information. *^+^ p* < .10, ** p* < .05*, ** p* < .01*, *** p* < .001.

|  |  | Outcome Variable | | |  |
| --- | --- | --- | --- | --- | --- |
| Covariate | Math Ability 2 | | Math Ability 3 | Math Ability 4 | |
| Sex | 0.03 | | 0.02 | -0.02 | |
| Age | 0.04 | | -0.02 | -0.10* | |


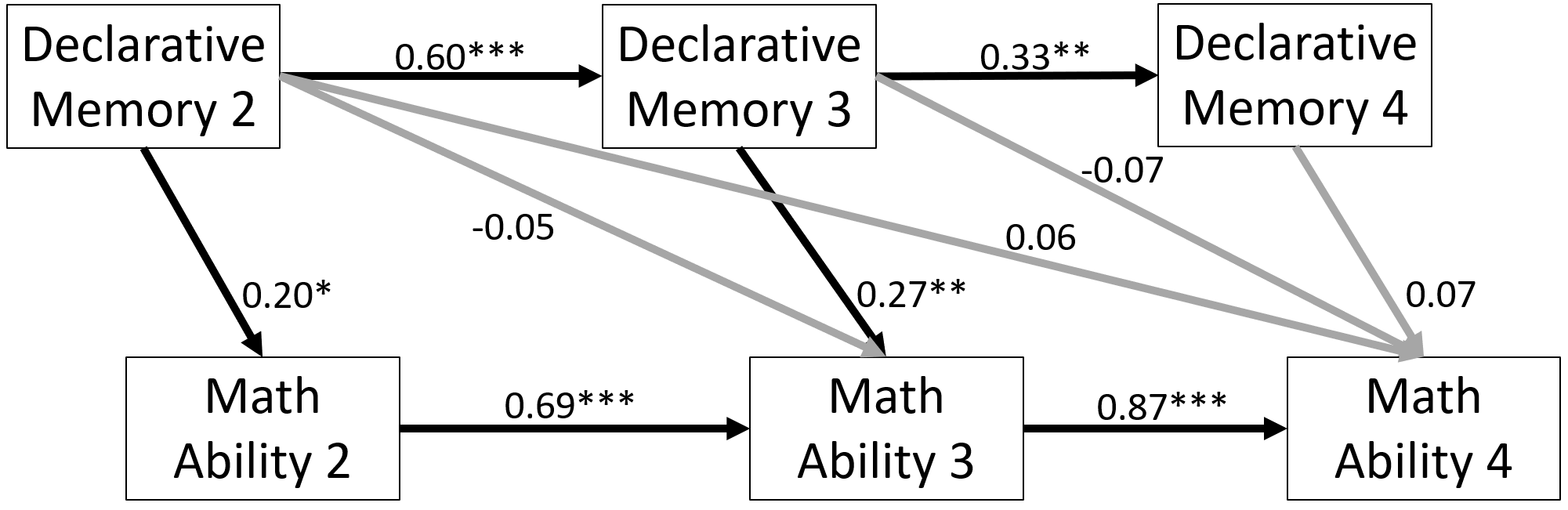


*Supplementary Figure 3.* Sample-restricted SEM without procedural memory. *CFI* = 0.87*, RMSEA*= 0.11, *AIC* = -626.05*, n-*adjusted *BIC* = -667.45, *N* = 61. See Note to Supplementary Figures 1 and 2 for more information. *^+^ p* < .10, ** p* < .05*, ** p* < .01*, *** p* < .001.

|  |  | Outcome Variable | | |  |
| --- | --- | --- | --- | --- | --- |
| Covariate | Math Ability 2 | | Math Ability 3 | Math Ability 4 | |
| Sex | 0.00 | | 0.02 | -0.01 | |
| Age | 0.07* | | -0.01 | -0.11* | |


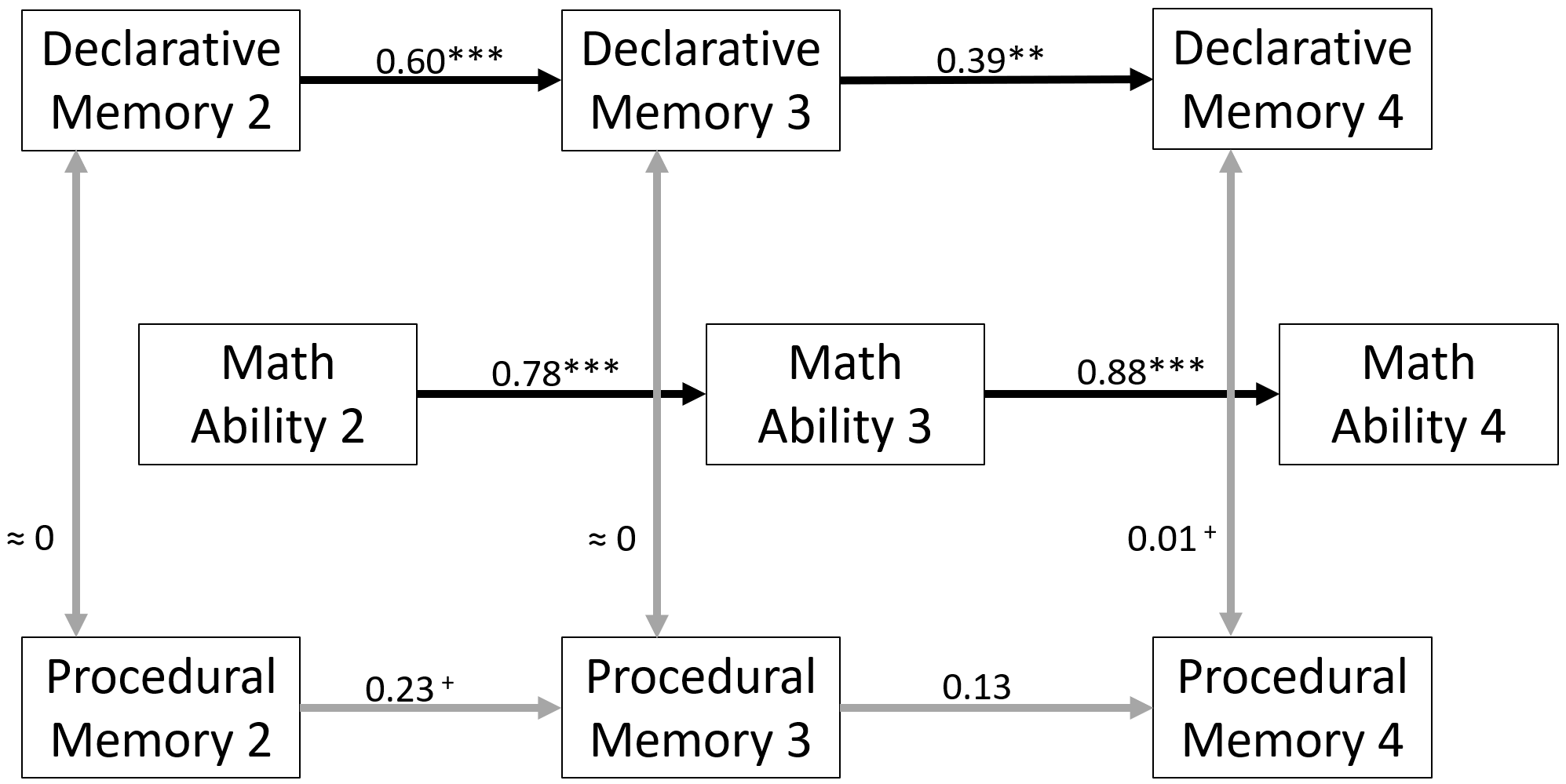


*Supplementary Figure 4.* SEM with autoregressive paths. *CFI* = .85*, RMSEA*= .07*, AIC* = -999.72*, n-*adjusted *BIC* = -1022.21, *N* = 109. The number at the end of each variable name refers to grade 2, 3, or 4. Lines with single arrows represent regressions, while double-arrows show covariances (i.e., between declarative and procedural memory). Significant paths are shown in black, while non-significant ones are gray. Unstandardized coefficients (*b*) are presented next to each path line. Data for the memory and math ability variables were transformed using Proportion of Maximum Scaling, and were person-centered for each grade; see Methods. Math ability regressions included sex and age in that grade as covariates, with these coefficients presented below the figure. *^+^ p* < .10, ** p* < .05*, ** p* < .01*, *** p* < .001.

Appendix 1 Mplus Code for doing correlation matrix-based SEM with longitudinal random intercepts

**Data:**

FILE is corr.txt;

*!This is the lower half of all pairwise-correlations without variables names; the variables names and order are determined in the “NAMES are” section below. The rounded correlations are available in Table 3.*

TYPE = Correlation;

NOBSERVATIONS = 109;

**Variable:**

NAMES are

sex

age2 age3 age4

math2 math3 math4

dm2 dm3 dm4

pm2 pm3 pm4;

USEVARIABLES are

math2 math3 math4

dm2 dm3 dm4

pm2 pm3 pm4;

**Analysis:**

ESTIMATOR is ML;

**Model:**

*!Random Intercepts*

ridm by dm2@1 dm3@1 dm4@1;

ripm by pm2@1 pm3@1 pm4@1;

rimath by math2@1 math3@1 math4@1;

*!Within Person Centering*

cdm2 by dm2@1;

cdm3 by dm3@1;

cdm4 by dm4@1;

cpm2 by pm2@1;

cpm3 by pm3@1;

cpm4 by pm4@1;

cmath2 by math2@1;

cmath3 by math3@1;

cmath4 by math4@1;

*!Measurement Error variances at 0*

math2-pm4@0;

*!Autoregressive Effects*

cmath3 on cmath2;

cmath4 on cmath3;

cdm3 on cdm2;

cdm4 on cdm3;

cpm3 on cpm2;

cpm4 on cpm3;

*!Cross Variable Effects*

cmath4 on cdm4 cdm3 cdm2;

cmath3 on cdm3 cdm2;

cmath2 on cdm2;

cmath4 on cpm4;

cmath3 on cpm3;

cmath2 on cpm2;

*!Covariances*

cdm2 with cpm2;

cdm3 with cpm3;

cdm4 with cpm4;

*!Fix the correlation between the individual factors and the other*

*!exogenous variables to zero (by default these would be estimated)*

ridm with cdm2@0 cpm2@0 cmath2@0;

ripm with cdm2@0 cpm2@0 cmath2@0;

rimath with cdm2@0 cpm2@0 cmath2@0;

*!To deactivate the random intercepts, fix their variances and covariance to !zero.*

!ridm@0;

!rimath@0;

!ridm WITH rimath@0;

**Output:**

*!SVALUES the model parameter results printed in the output so they can be !used as population values for the Monte Carlo power analysis.*

SVALUES;

*!TECH1 requests MPLUS to print the parameter specifications for all free !parameters. We specifically want the Parameter Specification Beta table to !identify the parameter identifier values to locate parameter variances and !covariances in TECH3 for the* *Monte Carlo Method for Assessing Mediation !(MCMAM)*

TECH1;

*!TECH3 requests the variance-covariance matrix for the parameters in the !model. The row and column values identify parameters based on the values in !the Beta table of TECH1, with the value where the identifiers are equal !being the parameter variance, and values where identifiers are different !being the covariances between parameters.*

TECH3;

Appendix 2 R Studio Code for performing the Monte Carlo Method for Assessing Mediation (MCMAM) from the correlation-based model parameters

**##################################################**

**#Setup**

**##################################################**

*#Clear Environment*

rm(list=ls())

*#Load Packages*

sapply(c('MplusAutomation', 'MASS', 'tidyverse', 'qdapRegex'), require, character.only = TRUE)

**##################################################**

**#Import Results**

**##################################################**

results<-list()

*#Load Model Output using MplusAutomation package. This file is designed to #look at multiple sets of results, namely the model with the deactivated #random intercept (ri@0) and on with it activated (ri).*

results$ri0<-readModels("ri@0", recursive=FALSE)

results$ri<-readModels("ri", recursive=FALSE)

**##################################################**

**#Defining Indirect Effect Pairs**

**##################################################**

*#These lists help to pair the names of the “a” path parameter, the “b” path, and the covariance between the parameters.*

{

e<-list()

e$a<-c("dm23", "dm34", "dc2", "dc3")

e$b<-c("dc3", "dc4", "math23", "math34")

e$cov<-c("ddc23", "ddc34", "dcc23", "dcc34")

}

**##################################################**

**#Define Monte Carlo Method for Assessing Mediation Function**

**##################################################**

*#This function will take the parameter coefficients, variances, and covariances from a model to generate a multivariate normal distribution of values (in this case 20,000) for the a and b paths in a mediation model; multiply the a and b values to get the indirect effect values; and finally find the median of indirect effect values in the simulated distribution along with the lower and upper 99^th^, 95^th^, and 90^th^ percentile values to serve as the confidence interval cutoffs. A seed is fixed in the function itself to ensure the same results are obtained.*

mcmam <- function(est_a, est_b, var_a, var_b, cov_ab) {

set.seed(58809)

pest=c(est_a,

est_b)

acov <- matrix(c(

var_a, cov_ab,

cov_ab, var_b

),2,2)

mcmc <- mvrnorm(20000,pest,acov,empirical=FALSE)

ab <- mcmc[,1]*mcmc[,2]

tmp<-

data.frame(

Lower99=format(quantile(ab,0.005), digits=4),

Lower95=format(quantile(ab,0.025), digits=4),

Lower90=format(quantile(ab,0.05), digits=4),

Median=format(quantile(ab, 0.50), digits=4),

Upper90=format(quantile(ab,0.95), digits=4),

Upper95=format(quantile(ab,0.975), digits=4),

Upper99=format(quantile(ab,0.995), digits=4),

row.names = NULL)

}

**##################################################**

**#Extract Information to calculate indirect effects**

**##################################################**

*#This loop extracts and sorts the necessary components for the MCMAM simulation from each model. It collects the parameter coefficients, locations of the coefficient beta values from the tech1 output to find the variances and covariances in tech3, and finally the parameter variances and covariances from tech3.*

ind<-list()

for(r in 1:length(results)) {

ind[[names(results[r])]]<-list()

ind[[names(results[r])]]$params<-list()

ind[[names(results[r])]]$loc<-list()

ind[[names(results[r])]]$covs<-list()

ind[[names(results[r])]]$est<-list()

ind[[names(results[r])]]$coord<-list()

ind[[names(results[r])]]$var<-list()

ind[[names(results[r])]]$cov<-list()

ind[[r]]$params<-results[[r]]$parameters$unstandardized

ind[[r]]$loc<-results[[r]]$tech1$parameterSpecification$X$beta

ind[[r]]$covs<-results[[r]]$tech3$paramCov

ind[[r]]$est$dm23<-ind[[r]]$params[ind[[r]]$params$paramHeader=="CDM3.ON" & ind[[r]]$params$param=="CDM2", "est"]

ind[[r]]$est$dm34<-ind[[r]]$params[ind[[r]]$params$paramHeader=="CDM4.ON" & ind[[r]]$params$param=="CDM3", "est"]

ind[[r]]$est$math23<-ind[[r]]$params[ind[[r]]$params$paramHeader=="CMATH3.ON" & ind[[r]]$params$param=="CMATH2", "est"]

ind[[r]]$est$math34<-ind[[r]]$params[ind[[r]]$params$paramHeader=="CMATH4.ON" & ind[[r]]$params$param=="CMATH3", "est"]

ind[[r]]$est$dc2<-ind[[r]]$params[ind[[r]]$params$paramHeader=="CMATH2.ON" & ind[[r]]$params$param=="CDM2", "est"]

ind[[r]]$est$dc3<-ind[[r]]$params[ind[[r]]$params$paramHeader=="CMATH3.ON" & ind[[r]]$params$param=="CDM3", "est"]

ind[[r]]$est$dc4<-ind[[r]]$params[ind[[r]]$params$paramHeader=="CMATH4.ON" & ind[[r]]$params$param=="CDM4", "est"]

ind[[r]]$coord$dm23<-ind[[r]]$loc["CDM3", "CDM2"]

ind[[r]]$coord$dm34<-ind[[r]]$loc["CDM4","CDM3"]

ind[[r]]$coord$math23<-ind[[r]]$loc["CMATH3", "CMATH2"]

ind[[r]]$coord$math34<-ind[[r]]$loc["CMATH4","CMATH3"]

ind[[r]]$coord$dc2<-ind[[r]]$loc["CMATH2","CDM2"]

ind[[r]]$coord$dc3<-ind[[r]]$loc["CMATH3","CDM3"]

ind[[r]]$coord$dc4<-ind[[r]]$loc["CMATH4","CDM4"]

ind[[r]]$var$dm23<-ind[[r]]$covs[ind[[r]]$coord$dm23, ind[[r]]$coord$dm23]

ind[[r]]$var$dm34<-ind[[r]]$covs[ind[[r]]$coord$dm34, ind[[r]]$coord$dm34]

ind[[r]]$var$math23<-ind[[r]]$covs[ind[[r]]$coord$math23, ind[[r]]$coord$math23]

ind[[r]]$var$math34<-ind[[r]]$covs[ind[[r]]$coord$math34, ind[[r]]$coord$math34]

ind[[r]]$var$dc2<-ind[[r]]$covs[ind[[r]]$coord$dc2, ind[[r]]$coord$dc2]

ind[[r]]$var$dc3<-ind[[r]]$covs[ind[[r]]$coord$dc3, ind[[r]]$coord$dc3]

ind[[r]]$var$dc4<-ind[[r]]$covs[ind[[r]]$coord$dc4, ind[[r]]$coord$dc4]

ind[[r]]$cov$ddc23<-ind[[r]]$covs[ind[[r]]$coord$dc3, ind[[r]]$coord$dm23]

ind[[r]]$cov$ddc34<-ind[[r]]$covs[ind[[r]]$coord$dc4, ind[[r]]$coord$dm34]

ind[[r]]$cov$dcc23<-ind[[r]]$covs[ind[[r]]$coord$math23, ind[[r]]$coord$dc2]

ind[[r]]$cov$dcc34<-ind[[r]]$covs[ind[[r]]$coord$math34, ind[[r]]$coord$dc3]

}

**##################################################**

**#Run MCMAM**

**##################################################**

*#This loop runs the MCMAM function through each designated indirect effect (named in the e$cov array) in each model to produce the confidence intervals of the distribution of simulated indirect effects.*

ci<-list()

for(r in 1:length(results)) {

for(i in 1:length(e$cov)) {

ci[[names(results[r])]][[paste0(e$cov[i])]] <-

mcmam(

est_a=ind[[r]]$est[[paste0(e$a[i])]],

est_b=ind[[r]]$est[[paste0(e$b[i])]],

var_a=ind[[r]]$var[[paste0(e$a[i])]],

var_b=ind[[r]]$var[[paste0(e$b[i])]],

cov_a=ind[[r]]$cov[[paste0(e$cov[i])]]

)

}

}

Appendix 3 Mplus Code for Monte Carlo power simulations of the longitudinal random intercept SEM using different number of subjects, missing data, and indirect effect size.

**Montecarlo:**

NAMES =

math2 math3 math4

dm2 dm3 dm4;

*!Number of subjects in our data*

NOBSERVATIONS = 109;

*!Increased number of subjects*

!NOBSERVATIONS = 200;

*!Seeds used for simulation with 109 subjects with missing data and .15*

*!indirect effects*

!Simulation 1

SEED = 75297223;

!Simulation 2

!SEED = 15994618;

*!Seeds used for simulation with 109 subjects with no missing data and .15*

*!indirect effects*

!Simulation 1

!SEED = 849511589;

!Simulation 2

!SEED = 149815113;

*!Seeds used for simulation with 200 subjects with no missing data and .15*

*!indirect effects*

!Simulation 1

!SEED = 78237526;

!Simulation 2

!SEED = 8495667;

*!Seeds used for simulation with 200 subjects with no missing data and .10*

*!indirect effects*

!Simulation 1

!SEED = 75297223;

!Simulation 2

!SEED = 891484198;

*!Total number of model replications.*

NREPS = 1000;

*!Missing Data Pattern and Probability in our data.*

*!Remove this section to simulate no missing data.*

PATMISS =

math2(0.156) math3(0.183) math4(0.266)

dm2(0.193) dm3(0.202) dm4 (0.275);

PATPROBS = 1;

**Analysis:**

ESTIMATOR=Bayes;

*!Maximum and minimum number of iterations to complete a replication*

BITERATIONS=100000 1000;

**Model:**

Model Population:

*!Means and Variances from Mplus model output that only estimated*

*!means and variances of variables, and printed their starting values*

*!as text using svalues in the Output section.*

[ math2@0.37062 ];

[ math3@0.54345 ];

[ math4@0.67882 ];

[ dm2@0.46159 ];

[ dm3@0.47840 ];

[ dm4@0.52318 ];

*!Variances*

math2@0.01440;

math3@0.02363;

math4@0.02999;

dm2@0.03103;

dm3@0.03010;

dm4@0.02912;

*!Random Intercepts*

ridm BY dm2@1;

ridm BY dm3@1;

ridm BY dm4@1;

rimath BY math2@1;

rimath BY math3@1;

rimath BY math4@1;

*!Within Person Centering*

cdm2 BY dm2@1;

cdm3 BY dm3@1;

cdm4 BY dm4@1;

cmath2 BY math2@1;

cmath3 BY math3@1;

cmath4 BY math4@1;

*!Measurement Error variances at 0*

!(will cause error if included in model population).

!math2@0;

!math3@0;

!math4@0;

!dm2@0;

!dm3@0;

!dm4@0;

*!Population Coefficients for indirect effects of .15*

cmath3 ON cmath2@.50;

cmath3 ON cdm3@.30;

cmath3 ON cdm2@.01;

cmath4 ON cmath3@.50;

cmath4 ON cdm4@.30);

cmath4 ON cdm3@.01;

cmath4 ON cdm2@.01;

cdm3 ON cdm2@0.50;

cdm4 ON cdm3@0.50;

cmath2 ON cdm2@0.30;

*!Population Coefficients for indirect effects of .10*

!cmath3 ON cmath2@.40;

!cmath3 ON cdm3@.25;

!cmath3 ON cdm2@.01;

!cmath4 ON cmath3@.40;

!cmath4 ON cdm4@.25;

!cmath4 ON cdm3@.01;

!cmath4 ON cdm2@.01;

!cdm3 ON cdm2@0.40;

!cdm4 ON cdm3@0.40;

!cmath2 ON cdm2@0.25;

*!Random Intercept Correlations*

rimath WITH ridm@0.30;

*!Fix the correlation between the individual factors and the other*

*!exogenous variables to zero (by default these would be estimated)*

ridm WITH cdm2@0;

ridm WITH cmath2@0;

rimath WITH cdm2@0;

rimath WITH cmath2@0;

*!Random Intercept & Centered Variable Variances*

ridm@0.34534;

rimath@0.62890;

cdm2@0.63377;

cdm3@0.56312;

cdm4@0.62814;

cmath2@0.39756;

cmath3@0.30290;

cmath4@0.28737;

**Model:**

*!Random Intercept*

ridm by dm2@1 dm3@1 dm4@1;

rimath by math2@1 math3@1 math4@1;

*!Within Person Centering*

cdm2 by dm2@1;

cdm3 by dm3@1;

cdm4 by dm4@1;

cmath2 by math2@1;

cmath3 by math3@1;

cmath4 by math4@1;

*!Measurement Error variances at 0*

math2-dm4@0;

*!Autoregressive Effects*

cmath3 on cmath2;

cmath4 on cmath3;

cdm3 on cdm2;

cdm4 on cdm3;

*!Cross Variable Effects*

cmath4 on cdm4;

cmath4 on cdm3 cdm2;

cmath3 on cdm3;

cmath3 on cdm2;

cmath2 on cdm2;

*!Fix the correlation between the individual factors and the other*

*!exogenous variables to zero (by default these would be estimated)*

ridm with cdm2@0 cmath2@0;

rimath with cdm2@0 cmath2@0;

*!Estimate Indirect Effects*

Model Indirect:

cmath3 IND cdm2;

cmath4 IND cdm3;

cmath4 IND cdm2;

**OUTPUT:**

*!Output equal-tail confidence intervals of model estimates and indirect*

*!effects from the Monte Carlo Replications*

CINTERVAL(eqtail);

**References**

1. Hooper, D., Coughlan, J. & Mullen, M. R. Structural Equation Modelling: Guidelines for Determining Model Fit. *Electron. J. Bus. Res. Methods* **6**, 53–60 (2008).

2. Bryant, F. B. & Satorra, A. Principles and Practice of Scaled Difference Chi-Square Testing. *Struct. Equ. Model. Multidiscip. J.* **19**, 372–398 (2012).

3. Satorra, A. & Bentler, P. M. A scaled difference chi-square test statistic for moment structure analysis. *Psychometrika* **66**, 507–514 (2001).

4. Bollen, K. A. & Stine, R. Direct and Indirect Effects: Classical and Bootstrap Estimates of Variability. *Sociol. Methodol.* **20**, 115–140 (1990).

5. MacKinnon, D. P., Lockwood, C. M. & Williams, J. Confidence Limits for the Indirect Effect: Distribution of the Product and Resampling Methods. *Multivar. Behav. Res.* **39**, 99–128 (2004).

6. Preacher, K. J. & Hayes, A. F. Asymptotic and resampling strategies for assessing and comparing indirect effects in multiple mediator models. *Behav. Res. Methods* **40**, 879–891 (2008).

7. Wolf, E. J., Harrington, K. M., Clark, S. L. & Miller, M. W. Sample Size Requirements for Structural Equation Models: An Evaluation of Power, Bias, and Solution Propriety. *Educ. Psychol. Meas.* **73**, 913–934 (2013).

8. Sideridis, G., Simos, P., Papanicolaou, A. & Fletcher, J. Using Structural Equation Modeling to Assess Functional Connectivity in the Brain: Power and Sample Size Considerations. *Educ. Psychol. Meas.* **74**, 733–758 (2014).

9. Koopman, J., Howe, M., Hollenbeck, J. R. & Sin, H.-P. Small sample mediation testing: Misplaced confidence in bootstrapped confidence intervals. *J. Appl. Psychol.* **100**, 194–202 (2015).

10. Enders, C. K. & Bandalos, D. L. The Relative Performance of Full Information Maximum Likelihood Estimation for Missing Data in Structural Equation Models. *Struct. Equ. Model. Multidiscip. J.* **8**, 430–457 (2001).

11. Hamaker, E. L., Kuiper, R. M. & Grasman, R. P. P. P. A critique of the cross-lagged panel model. *Psychol. Methods* **20**, 102–116 (2015).

12. Hamaker, E. L. How to run the RI-CLPM with Mplus. (2018).

13. Selig, J. P. & Preacher, K. J. Monte Carlo method for assesing mediation: An interactive tool for creating confidence intervals for indirect effects. (2008).

14. Thoemmes, F., Mackinnon, D. P. & Reiser, M. R. POWER ANALYSIS FOR COMPLEX MEDIATIONAL DESIGNS USING MONTE CARLO METHODS. *Struct. Equ. Model. Multidiscip. J.* **17**, 510–534 (2010).

15. Zhang, Z. Monte Carlo based statistical power analysis for mediation models: methods and software. *Behav. Res. Methods* **46**, 1184–1198 (2014).

1. Due to the complexity and time-intensity of simulating longitudinal random intercept SEMs, performing more than 1,000 replications per simulation ran the risk of an error occurring in calculating the DIC across models, which would entirely stop the simulation. We performed each simulation twice to see if the simulation results were consistent. [↑](#footnote-ref-1)
